# Boosting cisplatin chemotherapy by nanomotor-enhanced tumor penetration and DNA adducts formation

**DOI:** 10.1186/s12951-022-01622-3

**Published:** 2022-09-29

**Authors:** Lihua Xu, Kaixiang Zhang, Xing Ma, Yingying Li, Yajie Jin, Chenglin Liang, Yong Wang, Wendi Duan, Hongling Zhang, Zhenzhong Zhang, Jinjin Shi, Junjie Liu, Yunlong Wang, Wentao Li

**Affiliations:** 1 National Center for International Research in Cell and Gene Therapy, Sino-British Research Center for Molecular Oncology, Academy of Medical Sciences, Zhengzhou University, Zhengzhou, 450052 Henan China; 2 Department of Breast Surgery, Henan Provincial People’s Hospital, Zhengzhou, 450003 Henan China; 3 Henan Bioengineering Research Center, Zhengzhou, 450000 Henan China; 4 School of Materials Science and Engineering & Flexible Printed Electronic Technology Center, Harbin Institute of Technology (Shenzhen), Shenzhen, 518055 China; 5 School of Pharmaceutical Sciences, Zhengzhou University, Zhengzhou, 450001 China

**Keywords:** Cisplatin chemotherapy, Nanomotor, Tumor penetration, DNA adduct, Ion regulation

## Abstract

Despite many nano-based strategies devoted to delivering cisplatin for tumor therapy, its clinical benefits are compromised by poor tissue penetration and limited DNA adducts formation of the drug. Herein, a cisplatin loading nanomotor based janus structured Ag-polymer is developed for cisplatin delivery of deeper tissue and increased DNA adducts formation. The nanomotor displayed a self‐propelled tumor penetration fueled by hydrogen peroxide (H_2_O_2_) in tumor tissues, which is catalytically decomposed into a large amount of oxygen bubbles by Ag nanoparticles (NPs). Notably, cisplatin could elevate the intracellular H_2_O_2_ level through cascade reactions, further promote the degradation of Ag NPs accompanied with the Ag^+^ release, which could downregulate intracellular Cl^−^ through the formation of AgCl precipitate, thereby enhancing cisplatin dechlorination and Pt–DNA formation. Moreover, polymer can also inhibit the activity of ALKBH2 (a Fe^2+^-dependent DNA repair enzyme) by chelating intracellular Fe^2+^ to increase the proportion of irreparable Pt–DNA cross-links. It is found that deep tissue penetration, as well as the increased formation and maintenance of Pt–DNA adducts induced by the nanomotor afford 80% of tumor growth inhibition with negligible toxicity. This work provides an important perspective of resolving chemotherapeutic barriers for boosting cisplatin therapy.

**Graphical Abstract:**

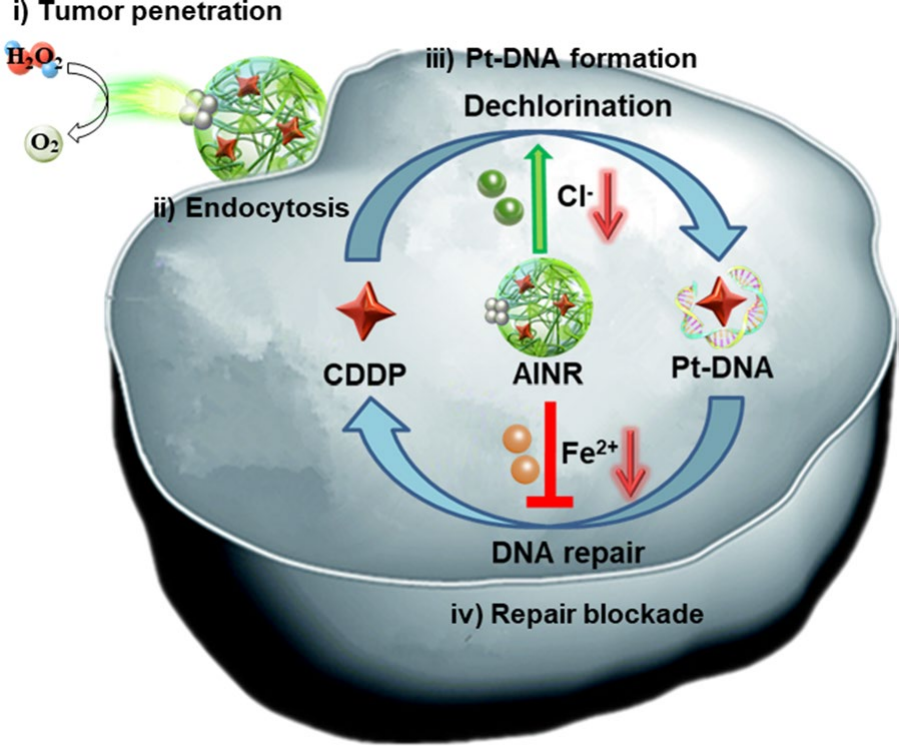

**Supplementary Information:**

The online version contains supplementary material available at 10.1186/s12951-022-01622-3.

## Introduction

Due to the antitumor broad-spectrum, cisplatin (cis-diamminedichloroplatinum [CDDP]) remains the frontline therapy against various cancers, whose main pharmacological effect is to inhibit DNA replication by forming Pt–DNA adducts, thus leading to apoptosis of tumor cells [1, 2]. However, its chemotherapeutic applications are generally limited by insufficient tumor chemotherapy response. Therefore, higher doses of CDDP are commonly used clinically to achieve the expected therapeutic effect, while causing severe nephrotoxicity, neurotoxicity, and other toxic side effects, thus limit its clinical benefits [3, 4]. It is of pressing need a design for enhancing therapeutic efficacy and reducing side effects of cisplatin.

Increased evidence indicating inefficient tumor chemotherapy response of cisplatin involve a multistep cascade process [5], including uneven tumor tissue distribution, reduced intracellular uptake, limited Pt–DNA adducts formation and enhanced self-repair ability of DNA in tumor cells. Copper transporter (Ctr1) was reported to be the main mediator to control cisplatin uptake, while its expression in tumor cells is always down-regulated [6]. The development of nanocarriers has revolutionized the cellular uptake way of small molecule drugs, greatly promoting the intracellular transport of drugs. [7] However, the dense tumor extracellular matrix (ECM) hampers the tissue penetration of nanomedicine and limits its therapeutic benefits [8, 9]. For instance, Doxil with a diameter of ≈100 nm has been reported to predominantly localize near the vasculature [10]; therefore, therapeutic drugs can be delivered only to peripheral cells of the tumor mass, resulting in limited therapeutic efficacy. There is an urgent need to develop nanocarriers for drug delivery in dense tumor tissues.

Notably, accumulated evidences have shown that less than 1% of CDDP internalized by tumor cells are able to form Pt–DNA adducts [11]. Generally, the activation of cisplatin involves a replacement of chloride ligands with water molecules [12]. The equated platinum complex can bind to guanine bases on DNA, forming intrastrand cross-links to destroy cancer cells [13]. Since chloridion ions inhibit the hydrolysis and the substitution of -OH to Cl ligand of cisplatin, low Cl^−^ media facilitates the reaction [14]. Apart from this, cisplatin-mediated DNA damage (Pt–DNA adducts) can activate subsequent the DNA damage response (DDR, maintaining human genome integrity) and DNA repair, that is to say, highly expressed DNA repair enzymes would rapidly excise Pt–DNA adducts and restore the genome sequence [15, 16]. Studies have found that ions, as coenzyme factors of multiple intracellular enzymes, play a key role in various life processes [17, 18]. ALKBH2, as a Fe^2+^-dependent dealkylation DNA repair protein, has been identified as the major repair enzyme to lesions in both ss- and ds-DNA induced by platinum based anticancer drugs, and the decrease of intracellular free Fe^2+^ would significantly inhibit its activity [19, 20]. Therefore, regulating the intracellular Cl^−^ and Fe^2+^ simultaneously will show great potential to perform an enhanced accumulation of Pt–DNA adducts in tumor cells. While, to the best of our knowledge, there is no report on the use of ion regulation strategies to improve the DNA adducts formation of cisplatin.

**Fig. 1:**
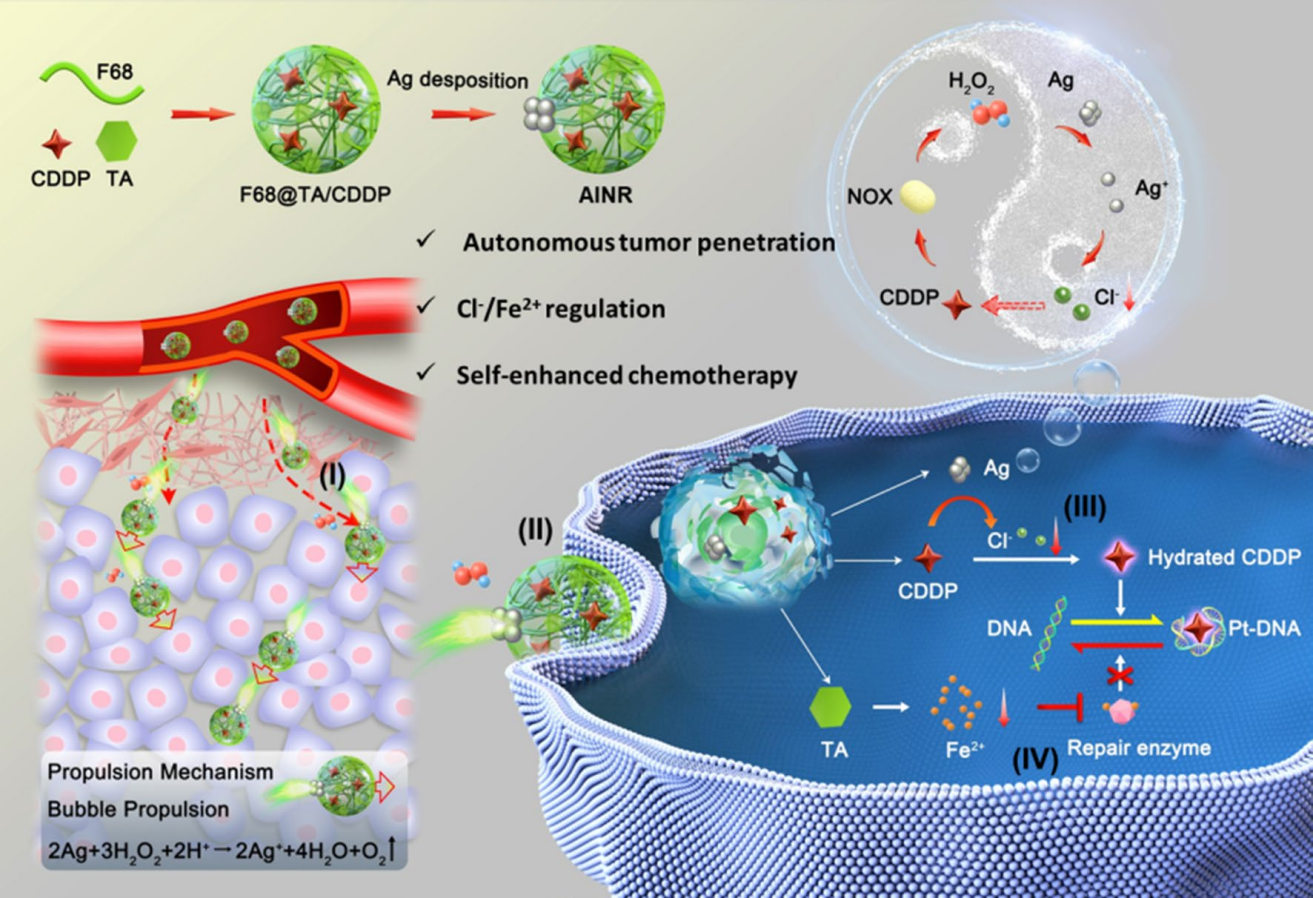
Schematic illustrations of construction and action mechanisms of AINR for boosting cisplatin chemotherapy. (I) AINR with Janus structures could penetrate into deeper tumor via gas-propelled autonomous movement fueled by H_2_O_2_ in tumor tissue; (II) AINR promote the uptake of cisplatin through nanoparticle endocytosis manner; (III) AINR could be oxidized into Ag^+^ under H_2_O_2_ in tumor cells, which down-regulating intracellular Cl^−^ through formation of AgCl precipitation, thus increasing the Pt–DNA adducts formation by promoting the dechlorination of cisplatin; (IV) AINR inhibits Pt–DNA excision repair by chelating intracellular Fe^2+^ to inhibit Fe^2+^-dependent DNA repair enzymes, finally realizing a self-augmented CDDP chemotherapy

Herein, a CDDP lading nanomotor with the H_2_O_2_-powered autonomous movement and intracellular dual-ion regulation, named asymmetric intracellular ion nanoregulator (AINR) that comprehensively harnessing the key factors involved in the cascade process of cisplatin chemotherapy, for boosting the efficacy of tumor treatment (Fig. 1). Specifically, we used tanic acid (TA) and poloxamer 188 (F68) as two building units for self-assembly as a nanocarrier for CDDP delivery. Besides, Ag nanoparticles (NPs) were in situ deposited on one side of CDDP loaded polymer to prepare an asymmetric nanoarchitecture (AINR). The janus structural AINR performed autonomous movement fueled by H_2_O_2_ in tumor tissues, which was catalytically decomposed into a large amount of oxygen (O_2_) bubbles by Ag NPs. The local concentration gradient of O_2_ bubbles could drive drug for deeper tumor penetration. And evenly distributed AINR are rapidly internalized by tumor cells through endocytosis. Subsequently, AINR cascade promotes accumulation of Pt–DNA adducts: Ag NPs in AINR could be rapidly oxidized to Ag^+^ under H_2_O_2_ in tumor cell [21, 22], which down-regulating intracellular Cl^−^ through formation of AgCl precipitation, thus increasing the Pt–DNA adducts formation by promoting the dechlorination of cisplatin; On the other hand, TA, a natural polyphenol, could act as an efficient chelating agent to reduce intracellular Fe^2+^, [23] thus enhanced the maintenance of Pt–DNA adducts by inhibiting Fe^2+^-dependent DNA repair enzymes. Notably, cisplatin could induce the production of H_2_O_2_ through specific activation of tumor overexpressed NADPH oxidases (NOXs) [24], in turn accelerates the down-regulation of intracellular Cl^−^, realizing a self-augmented CDDP chemotherapy. Taken together, the as-prepared AINR induce a robust cisplatin-based tumor chemotherapy through harnessing the cascade chemotherapeutic barriers.

## Materials and methods

### Materials and reagents

Tannic acid (TA), Pluronic® F68, cisplatin (CDDP) and Rhodamine B (RB) were purchased from Yuanye biotechnology company (Shanghai, China). Silver nitrate was obtained from aladdin reagent Co., Ltd. (Shanghai, China). Sodium Dodecyl Sulfonate (SDS) was bought from Sigma-Aldrich (USA). Phosphorylated H2AX (γH2AX) antibody was supplied by Cell Signaling Technology company. NOX4, and NAPDH antibody were purchased from Proteintech company. H_2_O_2_ fluorescent probe BES H_2_O_2_-Ac was got from Wako (Cat. #021-17801 (1 mg), Japan). MQAE probe and Fe^2+^ probe (Ferro Orange) were bought from Beyotime company (Shanghai, China) and Dong Ren company (Shanghai, China), respectively.

### Preparation of AINR

Prior to synthesis of AINR, F68@TA/CDDP was prepared according to previous literature with a little modification. In brief, F68 was dissolved in dimethyl formamide (DMF, 200 μL) with the final concentration of 20.0 mg/mL and TA was dissolved in DMF (200 μL) with the final concentration of 30.0 mg/mL. F68 and TA were mixed at molar ratio of 0.13. CDDP (8.00 mg) was dissolved by 400 μL of the mixture of F68 and TA solution. Afterwards, the mixture was added dropwise to the 40 mL of deionized water under vigorous stirring for 10 min. The resulting product (F68@TA/CDDP) was transferred into the dialysis bag (MWCO12,000-14,000) and immediately dialyzed against deionized water over 48 h to remove free TA and DMF. For AINR, 100 mL of dialyzed F68@TA/CDDP was added to 250 mL flask. Then sodium dodecyl sulfate (SDS, 40.0 mg) in 100 μL deionized water was added to the reaction system and stirred for 2 h at room temperature. After that, AgNO_3_ (30.0 mg) in 100 μL deionized water was involved and the reaction continued for 2 d at room temperature under vigorous stirring. Free SDS and AgNO_3_ were removed by dialysis.

To determinate the loading capacity of CDDP and Ag on AINR, ICP-MS was conducted. Prior to ICP-MS detection, 4 mL of AINR (containing 1 mg of F68@TA) was centrifugated at 12,000 rpm for 10 min and digested by 1 mL of concentrated nitric acid and 1 mL of 30% H_2_O_2_ overnight. The loading capacity of CDDP on F68@TA/CDDP (containing 1 mg of F68@TA) was also measured according to above method, and the drug loading efficiency was calculated with the following equation:

$${\text{Drug loading efficiency}}/\% = \frac{m(CDDP)}{{m(Nanoparticles) + m(CDDP)}}$$

in which, *m(CDDP)* represented the loading CDDP amount, and the *m(Nanoparticles)* represented the amount of  F68@TA or F68@TA@Ag.

For drug release experiment, 4 mL of F68@TA/CDDP (containing about 100 μg/mL CDDP) was obtained by centrifugation at 12,000 rpm for 10 min, and incubation time was set as 0.167, 0.5, 1, 2, 4, 6, 8, and 12 h. At different time points, the samples at different pHs (pH7.4, 6.5, and 5.0) were centrifugated at 12,000 rpm for 10 min, and the supernatant was used for CDDP detection by HPLC (Waters 2695) with the following chromatographic conditions: a symmetry C18 (250 mm × 4.6 mm, 5 µm); mobile phase, saline containing 0.003 M sodium heptane sulfonate; column temperature, 30 ℃; flow rate, 1.0 mL/min; and injection volume, 10 µL; detector, 2998 PDA; wavelength, 220 nm.

### pH-sensitive degradation of F68@TA/CDDP

4 mL of F68@TA/CDDP was centrifugated and resuspended in 1 mL of PBS with different pHs (pH7.4, 6.5, and 5.0) for different time. TEM and DLS were used to observe degradation behavior of  F68@TA/CDDP.

### *Ag NPs degradation and* *Ag*^+^*release induced by H*_*2*_*O*_*2*_

To investigate the disintegration of Ag nanoparticles on F68@TA@Ag responsive to H_2_O_2_, 1 mL of F68@TA@Ag (containing about 70 μg of Ag) was incubated with H_2_O_2_ in different concentration (0.01, 0.1 and 1 mM) at room temperature for 3 h, and TEM and DLS was conducted to observe the morphology and size of F68@TA@Ag NPs. The release of Ag^+^ in all the supernatant was measured by ICP-MS. Moreover, UV–vis spectrophotometer was used to detect the changes of the characteristic peak at 470 nm.

### Self-propelled movability of F68@TA@Ag fueled by H_2_O_2_

For the generation efficiency of O_2_, a drop of 30% H_2_O_2_ was added into the F68@TA@Ag solution. The phenomenon was recorded for 1 min. For the movability, the F68@TA@Ag was placed in a petri-dish filled with aqueous solution containing varied concentration of H_2_O_2_ (0, 2.5, 5, 10, 25, and 50 mM). A cover lip was placed onto the well of the petri-dish to seal the well and minimize the drifting effect. Each video of the movement of nanoparticles over 20 s was recorded with a CCD camera at a frame rate of about 20 fps under phase contrast. An inverted optical microscopy (LSM510, Zeiss) with 40 × objective was used for the observation of the movement. Then, the video was analyzed by software Image J for the tracking and speed analysis. The mean square displacement (MSD) of F68@TA@Ag was computed based on the tracked motor trajectory and the following equation:

$${\text{MSD}}(\Delta {\text{t}}) = \langle (\vec r({\text{t}} + \Delta {\text{t}}) - \vec r({\text{t}}))^{2}\rangle {\text{t}}$$

where $\vec r$ is velocity calculated from the tracking data [25].

The diffusion coefficient (Dt) is obtained by fitting the data to the plot MSD(Δt) versus time interval, according to the following equation, MSD(Δt) = 4·Dt·Δt, which holds valid at low time intervals and for small particles with low rotational diffusion [26]. For each condition, 30 particles were analyzed to obtain the statistic results.

### *Fe*^*2*+^*chelation by F68@TA *in vitro

Fe^2+^ chelation ability was determined by the colorimetry of Fe^2+^ and 1,10-phenanthroline. Briefly, F68@TA containing TA (1 μmol) was mixed with Fe standard solution in different concentration at 37℃ overnight. Next the mixture (1 mL) was transferred into dialysis bag (Mw1000), which was dialyzed in 20 mL of deionized water for 24 h. Then 10 mL of dialysate was pipetted to a 50 mL of centrifugation tube, and hydrochloric acid (1 mL, 10%) was added for Fe^3+^ reduction. After sufficient mixing, 1, 10-phenanthroline (2 mL, 0.15%) and sodium acetate (5 mL, 1 M) was added, and the final volume was fixed to 50 mL by deionized water. Finally, optical density of the samples was read by microplate spectrophotometer (H1 Synergy, Biotek) at 510 nm.

### F68@TA mediated DNA repair enzyme inhibition

The enzyme inhibition studies were performed with the recombinant ALKBH2 proteins (1.6 μΜ) and single strain DNA substrates (10 μΜ) in the 10 μL reaction buffer. The conditions were Tris·HCl (50 mM at pH7.5), α-ketoglutarate (50 μΜ), L-Ascorbate (2 mM), MgCl_2_ (10 mM), FeSO_4_ (0, 100, 150, or 200 μM)) for 2 h at 37 ˚C. In this reaction system, 1 μL TA or F68@TA was added to chelate Fe^2+^. TA was set as 1 mM. Given the fact that MgCl_2_ can precipitate with TA and Fe^2+^ in the Tris HCl buffer, the reaction was performed first to remove the precipitation of MgCl_2_, TA and Fe^2+^, and then DNA repair process was carried out. To test the enzyme inhibition, methylation-sensitive restriction enzymes DpnII was used to shear repaired DNA at 37 °C for 3 h, and the non-denatured 12% TBE-PAGE gel electrophoresis was conducted.

DpnII shear specificity was also studied. Methylated DNA (1 μΜ) or unmethylated DNA (1 μΜ) were incubated with or without DpnII (1000 units, NEB) for 3 h. The DNA sequences of unmethylated DNA and methylated DNA were shown in Additional file 1: Table S1.

### *Elevated intracellular H*_*2*_*O*_*2*_* induced by CDDP*

4T1 cells were seeded in a 6-well plate with the density of 3 × 10^5^ cells per well, and incubated overnight. Next, fresh culture medium with CDDP in different concentration (0, 0.5, 1, 2 and 4 μg/mL) was added. After 18 h of coincubation, H_2_O_2_ specific fluorescent probe BES-H_2_O_2_-Ac (50 μM, Wako company) was used to indicate intracellular H_2_O_2_ level. H_2_O_2_ generation induced by CDDP was observed by fluorescence microscope at excitation wavelength of 488 nm and optimal emission wavelength of 525 nm. Mean fluorescence intensity was analyzed by Image J.

### The degradation of F68@TA@Ag in 4T1 cells

Biological electron microscopy was conducted for intuitive observation of the degradation of Ag nanoparticles on F68@TA@Ag in 4T1 cells. In brief, 4T1 cells were seeded in the culture dishes overnight, and then treated with F68@TA, F68@TA@Ag, and AINR (containing 1 μg/mL of CDDP) for 5 h. After incubation, cells were collected fixed by glutaraldehyde, embedded by resin and sliced for observation via Bio-TEM (HT7800).

### *Detection of intracellular Cl*^*−*^* and Fe*^*2*+^*in 4T1 cells*

4T1 cells (3 × 10^5^ cells) were seeded in confocal petri dish. After adherence, cells were treated with different formulation (CDDP, F68@TA, F68@TA@Ag, and AINR) for 18 h. In this experiment, the equivalent concentration of CDDP was set as 1 μg/mL. At the end of treatment, cells were washed by PBS, and stained with MQAE probe (5 mM, Beyotime Biotech) for 1 h at 37 ℃. CLSM was used to observe the intracellular fluorescence with the excitation of 405 nm and emission wavelength of 420–480 nm. Mean fluorescence intensity was analyzed by Image J.

For Fe^2+^ detection, cells were stained with Ferro Orange probe (1 μM, Tongren Biotech) for 30 min at 37 ℃. CLSM was used to observe the intracellular fluorescence with the excitation of 552 nm and emission wavelength of 570–620 nm. Mean fluorescence intensity was analyzed by Image J.

### *Cytotoxicity of AINR *in vitro

4T1 cells were seeded in 96-well plate with the density of 5 × 10^3^/well. After adherence, cells were treated by free CDDP, F68@TA/CDDP and AINR with different concentration of CDDP for 24 h. 10 μL of CCK-8 was added to each well for another 3 h incubation. OD450 was read by microplate reader.

### Detection of Pt–DNA adducts content in 4T1 cells

4T1 cells were seeded in the culture dishes overnight, and then treated with different formulations (F68@TA, F68@TA@Ag, CDDP, F68@TA/CDDP, and AINR) for 24 h. The equivalent concentration of CDDP was 1 μg/mL. After incubation, cells were lysed by 0.2 mL of DNA lysis buffer containing 100 mM Tris–HCl (pH8.0), 25 mM EDTA (pH8.0), 500 mM NaCl, 1% SDS and proteinase K (with the final concentration of 100 μg/mL) at 45 ℃ for 2 h. RNase A (20 μg/mL) was used to degrade intracellular RNA. Subsequently, DNA was extracted using a phenol–chloroform method and quantified. 100 μg of extracted DNA in each group was digested by concentrated nitric acid and 30% H_2_O_2_. Pt content in DNA was measured by ICP-MS.

### DNA damage detection

For western blotting assays, 4T1 cells were seeded in the culture dishes overnight, and then treated with F68@TA, F68@TA@Ag, CDDP, F68@TA/CDDP, and AINR) for 24 h. The equivalent concentration of CDDP was 1 μg/mL. After incubation, cells were collected and lysed for total proteins. BCA kit (solarbio) was used to quantify total amount of proteins in each group. After gel electrophoresis, proteins were transferred into PVDF membrane for 2 h at 300 mA. Then PVDF membrane was blocked by 5% skim milk for 2 h at room temperature. After that, primary antibody containing GAPDH antibody (1:10,000, proteintech) and γH2AX antibody (1:1000, CST) were incubated at 4 ℃ overnight, and second antibody (1:1000, proteintech) was incubated at room temperature for 1 h. At last PVDF membrane was exposed by gel imager. Relative gray value was analyzed by Image J.

For comet assay, 4T1 cells were seeded in a 6-well plate, and the treatment was same as that in western blot assay. The detail process was performed according to the reported method. Tail rate was analyzed by casp software.

### Penetration of F68@TA@Ag in 3D multicellular sphere model and tumor tissues

To build a 3D multicellular sphere model (MCS), 100 μL of autoclaved 1% agarose was added in each well of the 96-well plate immediately, and cooled at room temperature. Then 100 μL of 4T1 cells (5 × 10^3^/well) were seed in 96-well plate and shook to hold cells together. After 4 d, 50 μL of fresh medium was added for cell proliferation. The MCS was successfully constructed at 7 d. Prior to drug treatment, 100 μΜ H_2_O_2_ was added to the MCS for 12 h. Then 100 μL of fresh medium containing F68@TA/RB or F68@TA@Ag/RB (RB: 10 μg/mL) was added after aspiration of original medium. After 12 h incubation, tumor spheres were washed by PBS, and stained by DAPI. CLSM was used to observe fluorescence distribution at each focal plane every 10 μm.

To observe the deep penetration ability of F68@TA and F68@TA@Ag in tumor tissues. F68@TA/RB and F68@TA@Ag/RB (RB: 20 μg/mL) were injected into 4T1 cells bearing mice by tail vein. After 24 h, tumor tissues were exfoliated for immunofluorescent staining. FITC-CD31 antibody was used to label tumor vessels.

### In vivo* biodistribution analysis*

IR783, a kind of water soluble near infrared dyes was used to visualize the biodistribution of F68@TA@Ag. F68@TA@Ag/IR783 or free IR783 (30 μg/mL) was administrated to the tumor bearing mice once. The images were captured at different time points (0.5, 1, 2, 5, 8, 12, 24, and 48 h) by MS FX live animal imaging system in vivo (Bruker). After 48 h, tumors and other main organs (heart, liver, spleen, lung, and kidney) in each group were exploited and imaged.

### In vivo* antitumor efficiency and biosafety studies*

4T1 cells-bearing BALB/C mice with an average tumor volume of 200 mm^3^ were randomly divided into six groups (five mice per group) and administered with (1) saline, (2) F68@TA, (3) F68@TA@Ag, (4) CDDP, (5) F68@TA/CDDP, and (6) AINR via tail vein every other day for 2 weeks, respectively. The equivalent dosage of CDDP was set as 2 mg/kg. During treatment, tumor volume was measured by a caliper and body weight of all mice was recorded.

After treatment, tumor tissues in all groups were exfoliated for hematoxylin and eosin (H&E) staining and terminal deoxynucleoitidyl transferase-mediated dUTP nick, labeling (TUNEL) staining.

To assess the biosafety of AINR, blood was also collected for liver and kidney function detection, and main organs (heart, liver, spleen, lung, and kidney) were exfoliated for hematoxylin and eosin (H&E) staining.

### Statistical analysis

Data are expressed as mean ± standard deviation (SD). Statistical analysis was assessed using one-way ANOVA test. A value of p < 0.05 was considered statistically significant.

## Results and discussions

### Fabrication and characterization of AINR

**Fig. 2:**
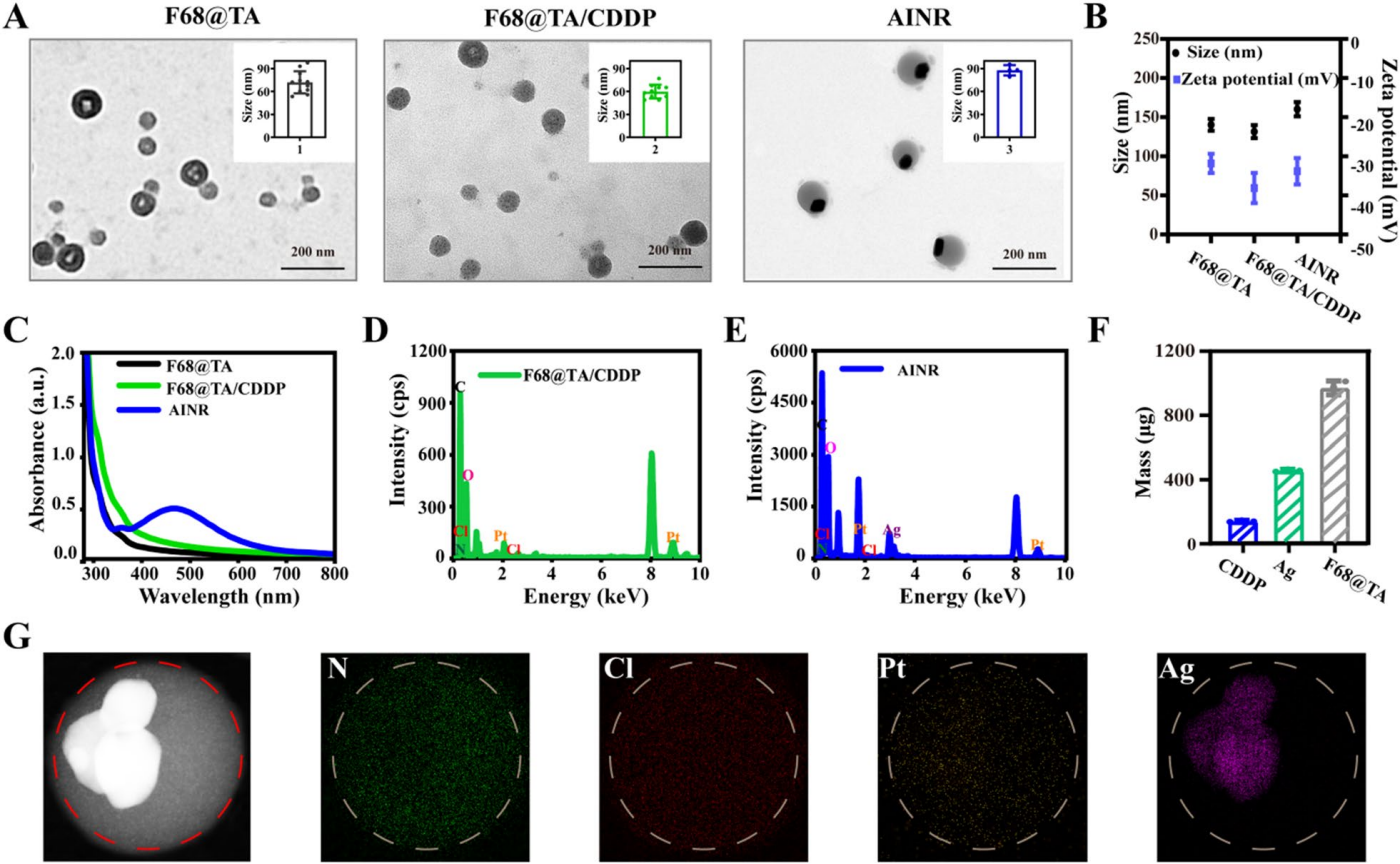
Characterization of AINR. **A**–**C** Representative TEM images, DLS analysis and UV–vis spectrum of F68@TA, F68@TA/CDDP, and AINR. Graphs in TEM images were statistical analysis for diameters of nanoparticles by Image J. **D**, **E** EDS analysis of **D** F68@TA/CDDP and **E** AINR. **F** The amount of CDDP, Ag, and F68@TA in AINR. (n = 3). **G** TEM mapping of AINR

In this study, the asymmetric ion nanoregulator (AINR) was prepared by encapsulation of CDDP in F68@TA polymer with about 8.5% of loading efficiency, and then in situ deposition of Ag NPs on the surface of polymer. Firstly, the self-assembled F68@TA polymer was prepared, and the devoted mass ratios of F68/TA were also optimized (Additional file 1: Figure S1). It can be seen from Transmission Electron Microscope (TEM) images (Fig. 2A) that the polymer was spherical hollow structures with a diameter of about 70 nm. The CDDP loaded polymer showed a solid sphere with a size of approximately 60 nm. Finally, AINR was prepared and optimized by adjusting the amount of AgNO_3_ (Additional file 1: Figure S2). Compared with CDDP loaded polymer, AINR demonstrated an asymmetric structure with Ag NPs on one side, and the diameter was approximately 80 nm, attesting the successful deposition of Ag NPs (Fig. 2A). The hydrodynamic size distributions and zeta potentials of above nanoparticles were further analyzed via dynamic light scattering (DLS, Fig. 2B and Additional file 1: Figure S3), indicating a good dispersity in the water solution. As shown in Fig. 2C, UV–vis spectrum of F68@TA/CDDP was unchanged in spite of CDDP loading, while there was a characteristic peak of Ag NPs at about 470 nm for AINR. Energy Disperse Spectroscopy (EDS) was further conducted to certify CDDP loading and Ag existence (Fig. 2D and E). From elemental mapping images of AINR, the Ag element was mainly distributed on one side of the nanostructure, while the elements N, Cl and Pt were homogenously distributed (Fig. 2G), showing a typical asymmetric structure. Besides, the loading capacity of CDDP and Ag reached 8.5% and 28.5%, as revealed by the analyses with Inductively Coupled Plasma Mass Spectrometry (ICP-MS) (Fig. 2F). Prior to ICP-MS detection, 4 mL of F68@TA@Ag/CDDP (containing 1 mg of F68@TA) was centrifugated and digested by 1 mL of concentrated nitric acid and 1 mL of 30% H_2_O_2_ overnight. Meanwhile, the loading capacity (12.8%) of CDDP in F68@TA nanoparticles was also conducted for further studies. All above results confirmed that AINR was successfully prepared.

### *pH-dependent CDDP release and H*_*2*_*O*_*2*_*-dependent Ag*^+^*release.*

**Fig. 3:**
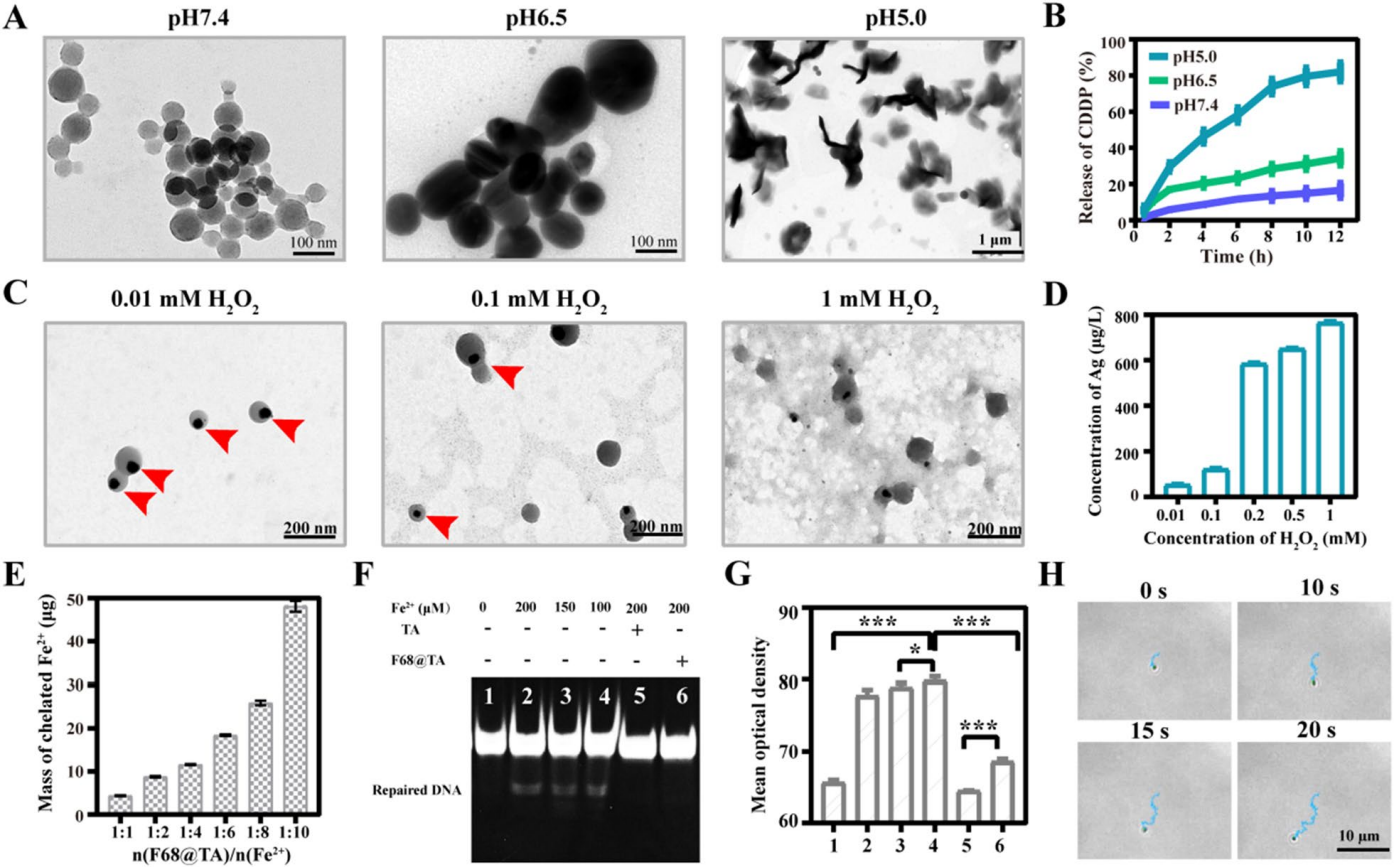
pH sensitivity, Fe^2+^ complexation, and movability of AINR in vitro. **A** TEM images of F68@TA/CDDP at different pHs for 1 h. **B** Release profile of CDDP at different pHs (n = 3). **C** TEM images of F68@TA@Ag after treated by different concentration of H_2_O_2_ for 3 h. **D** Release profile of Ag^+^ after treatment with different concentration of H_2_O_2_ for 3 h (n = 3). **E** Fe^2+^ content chelation by F68@TA at different molar ratio of F68@TA and Fe^2+^ after 12 h chelation (n = 3). The molar weight of F68@TA was set as 1 μmol which was calculated by TA in F68@TA. **F** TBE-PAGE gel electrophoretogram of m1A DNA repair efficiency incubated with TA or F68@TA under different concentration of Fe^2+^ and **G** semi-quantitative analysis of repaired DNA. **H** Snapshots of a typical trajectory at 25 mM H_2_O_2_. The statistical analysis was performed by one-way ANOVA analysis. (n = 4, *p < 0.05, ** *p* < 0.01, *** p < 0.001)

Furthermore, pH-dependent swelling and CDDP release behavior of CDDP loaded polymer were detected, respectively. The morphology of CDDP loaded polymer at pH 6.5 remained little changed but the diameter increased compared with that at pH 7.4. However, CDDP loaded polymer was swollen and even partially broken at pH 5.0, and the diameter increased significantly to ~ 600 nm (Fig. 3A). The weak acidic mediated disassembly of CDDP loaded polymer was also confirmed by the decrease of light transmittance (Additional file 1: Figure S4). The corresponding size distributions of CDDP loaded polymer were about 100 nm, 130 nm and 900 nm at different pHs even within 1 h (Additional file 1: Figure S5). Meanwhile, the release of CDDP from CDDP loaded polymer was measured by High Performance Liquid Chromatography (HPLC) (Fig. 3B). The cumulative release rates of CDDP were about 16.5%, 34.3% and 82.0% after 12 h-incubation at pH 7.4, pH 6.5 and pH 5.0, respectively, laying the foundation for CDDP release in acid lysosomes.

Dechlorination of CDDP is the prerequisite for the formation of Pt–DNA adducts. As referred above, the level of Cl^−^ is negatively correlated to the dechlorination efficiency of CDDP. Here the impact of Cl^−^ on hydration of CDDP was studied by HPLC (Additional file 1: Figure S6), and the results confirmed that the hydration of CDDP was effectively enhanced by the reduction of Cl^−^. The precipitation reaction of Ag^+^ and Cl^−^ can accelerate hydration of CDDP through efficient consumption of Cl^−^. A previous study reported that Ag NPs can be disintegrated to Ag^+^ by H_2_O_2_. [27] The equation is 2Ag + 3H_2_O_2_ + 2H^+^ → 2Ag^+^ + 4H_2_O + O_2_↑. H_2_O_2_-mediated dissolution of Ag NPs in F68@TA@Ag was first observed by TEM (Fig. 3C), Ag NPs were almost completely dissolved by incubation with 1 mM of H_2_O_2_ for 3 h, partially with 100 μM of H_2_O_2_ and barely with 10 μM of H_2_O_2_. The changes of color and absorbance at about 470 nm of F68@TA@Ag solution with the treatment of different concentration of H_2_O_2_ further confirmed this result (Additional file 1: Figure S7).

Furthermore, the generation of Ag^+^ from F68@TA@Ag was measured by ICP-MS (Fig. 3D). With the increase of H_2_O_2_ from 100 μM to 1 mM, the concentration of Ag^+^ increased 8-folds. Notably, the Ag^+^ concentration in supernatant dramatically increased 5-folds accompanied by the concentration of H_2_O_2_ raised from 100 to 200 μM. It is acknowledged that the concentration of H_2_O_2_ in tumor tissues is about 100 μM. [21] Therefore, in spite of partial dissolution in tumor extracellular matrix (ECM), most of Ag NPs can be internalized by tumor cells. Ag NPs on the constructed F68@TA@Ag can be disintegrated into Ag^+^ to react with intracellular Cl^−^ in tumor cells with high level of H_2_O_2_.

### *Fe*^*2*+^*chelation mediated activity inhibition of DNA repair enzymes*

Besides enhancing the formation of Pt–DNA by reducing Cl^−^, inhibiting the activity of DNA repair enzymes by reducing Fe^2+^ is essential to maintain Pt–DNA adducts. TA, containing 30 hydroxyl groups, possesses excellent complexing ability with Fe ions. The Fe^2+^ chelation ability of F68@TA was explored (Fig. 3E). As the molar ratio of F68@TA to Fe^2+^ varied from 1:1 to 1:10, the chelated Fe^2+^ increased 10-folds, possessing a remarkable potential to block the combination of DNA repair enzymes and Fe^2+^. To confirm our assumption, the DNA repair activity of ALKBH2 (a kind of DNA repair enzymes) incubated with TA or F68@TA was detected by TBE-PAGE gel electrophoresis via the shear efficiency of DNA restriction endonuclease DpnII (detailed methods in the Supporting Information). As seen from Additional file 1: Figure S8, DpnII could selectively shear the repaired DNA rather than damaged DNA (e.g. methylated DNA). Given Fe^2+^ dependence nature of ALKBH2, DNA was not sheared without Fe^2+^ (Fig. 3F and 3G). In contrast, when Fe^2+^ was added and incubated, a significant recovery of ALKBH2 activity was observed, while the DNA repair ability of ALKBH2 was inactive again when incubating with TA or F68@TA, similar with the group without Fe^2+^, indicating excellent Fe^2+^ chelation ability and DNA repair inhibition ability of TA and F68@TA.

### Self-propelled movability fueled by H_2_O_2_ in vitro

Interestingly, during the experiment of the dissolution process of Ag NPs mediated by H_2_O_2_, we observed the formation of bubbles (Additional file 2: Video S1). After analysis, the potential reason is that the reaction of Ag NPs and H_2_O_2_ produces a large amount of O_2_ (2Ag + 3H_2_O_2_ + 2H^+^ → 2Ag^+^ + 4H_2_O + O_2_↑). Since Ag NPs are located on the side of AINR, we tested whether the preparation has movability propelled by the produced O_2_. Here we recorded motion behaviors through time-dependent tracking process with different concentration of H_2_O_2_. Time-dependent trajectory of the F68@TA@Ag with 25 mM of H_2_O_2_ up to 20 s was given in Fig. 3H and typical tracking trajectories of F68@TA@Ag in different concentration of H_2_O_2_ were presented in Additional file 1: Figure S9A and Additional file 3: Video S2. Due to the small diameter of ~ 100 nm, Brownian movement has strong influence on the movement of F68@TA@Ag according to the Stokes–Einstein equation and we investigated the enhanced Brownian movement by a mean-square-displacement (MSD) analysis. An increased slope of the average MSD showed a fuel concentration-dependent enhanced diffusion (Additional file 1: Figure S9B). Subsequently, we calculated the diffusion coefficients of F68@TA@Ag from the slop of MSD (MSD = 4.Dt.ΔT). The diffusion coefficient increased from 0.28 ± 0.18 µm.s^−1^ to 1.46 ± 0.29 µm.s^−1^ without H_2_O_2_ and with 50 mM of H_2_O_2_ respectively (Additional file 1: Figure S9C), which confirmed more and more violent ballistic motion with the increase of H_2_O_2_ level, suggesting the potential for deeper tumor penetration for the efficient ion regulation in tumor cells.

### Cellular uptake and lysosomal escape assay.

**Fig. 4:**
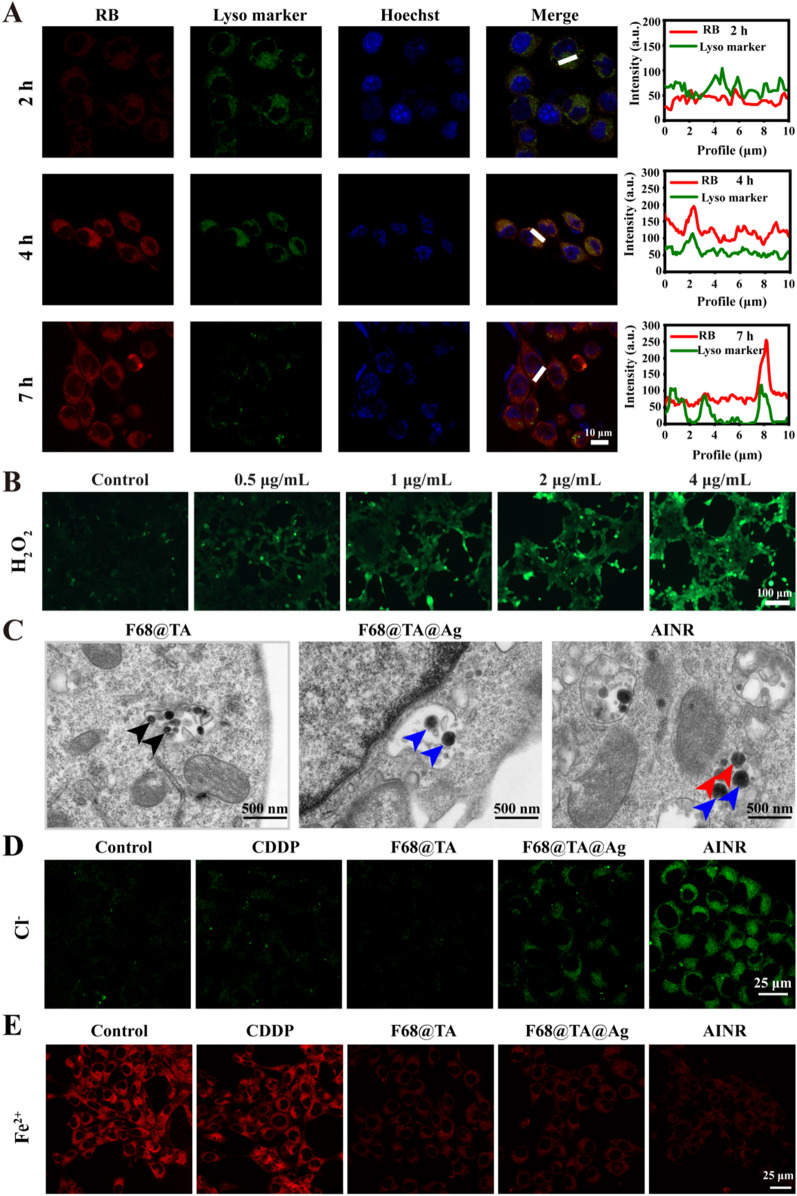
Intracellular distribution and ion regulation of AINR in 4T1 cells. **A** CLSM images and co-localization analysis of 4T1 cells after treatment with RB loaded polymer for different time. RB (red color) was used to mark F68@TA polymer, and lyso marker (green color) was used to indicate lysosomes. **B** Fluorescence images of 4T1 cells treated with different concentration of CDDP for 18 h. After treatment, cells were stained with H_2_O_2_ green probe (50 μΜ) for 30 min at 37 ℃. **C** Biological electron microscopy images of 4T1 incubated with F68@TA polymer, F68@TA@Ag and AINR for 5 h. Black arrow: polymer, blue arrow: F68@TA@Ag, and red arrow: F68@TA@Ag after dissolution of Ag NPs. **D** CLSM images of 4T1 cells stained by MQAE probe (5 mM for 1 h at 37 ℃) to mark intracellular Cl^−^ after treatment with CDDP, polymer, F68@TA@Ag and AINR for 18 h. Excitation wavelength: 405 nm and Emission wavelength: 420–470 nm. **E** CLSM images of 4T1 cells stained by Fe^2+^ probe (1 μM for 30 min at 37 ℃) to mark intracellular free Fe^2+^ after treatment with CDDP, F68@TA, F68@TA@Ag and AINR for 18 h. Excitation wavelength: 552 nm and Emission wavelength: 570–620 nm

Prior to evaluating the anti-tumor effect of AINR, we investigated the cellular uptake behavior of the polymer towards 4T1 cells. RB (Rhodamine B) was loaded into the polymer (Additional file 1: Figure S10). Flow cytometry and confocal laser scanning microscope (CLSM) were conducted for analysis of intracellular fluorescence intensity at different incubation time (Additional file 1: Figure S11 and S12). Fluorescence intensity of RB increased rapidly with incubation until 4 h, and the fluorescence intensity at 4 h was up to the saturated intake. Then the intracellular distribution of RB loaded polymer was explored by CLSM (Fig. 4A). A weak red fluorescence was found at 2 h incubation, and co-localization rate of red fluorescence (RB) and green fluorescence (lysosomes) was low, whereas an obvious increase of co-localization rate was observed after 4 h incubation, demonstrating that nanoparticles were located in the lysosomes at 4 h after internalization. The co-localization rate decreased and green fluorescence was weak after 7 h incubation, which may be attributed to swelling of the polymer at low pH. Based on this, it could be concluded that the polymer could escape quickly and release the loaded CDDP.

### *Intracellular Cl*^*−*^*/Fe*^*2*+^*regulation*

Dechlorination of CDDP is the key to form Pt–DNA adducts, which is regulated by the level of intracellular Cl^−^. Ag NPs on AINR are oxidized by H_2_O_2_ to release Ag^+^ for down-regulating intracellular Cl^−^. Therefore, the concentration of H_2_O_2_ is a vital factor for generation of Ag^+^. Previous research reported that CDDP can mediate activation of NOXs, which triggers O_2_ to produce abundant superoxide anion (O_2_^•−^), further to be transformed by superoxide dismutase (SOD) enzyme to form H_2_O_2_. [24] CDDP itself was a promising agent to raise intracellular H_2_O_2_. To prove the characteristic, 4T1 cells were treated with different concentration of CDDP, and stained by a H_2_O_2_ specific probe. Intracellular green fluorescence increased with the addition of CDDP, indicating that CDDP facilitated H_2_O_2_ generation and the process followed a concentration-dependent manner (Fig. 4B, and Additional file 1: Figure S13). CDDP mediated dissolution of Ag NPs on AINR was monitored by bio-TEM (Fig. 4C), and Ag NPs attached on F68@TA@Ag (blue arrows) were hardly any dissolved at 5 h of incubation, while disappeared in some AINR (red arrows), indicating that Ag NPs on AINR could be dissolved benefiting from CDDP mediated H_2_O_2_ production. Subsequently, we explored whether AINR could reduce intracellular Cl^−^ with the help of CDDP. Here MQAE probe was used to indicate intracellular Cl^−^, and the fluorescence intensity was negatively correlated to the level of Cl^−^ (Fig. 4D, and Additional file 1: Figure S14). Intracellular Cl^−^ was not significantly changed with CDDP treatment alone. However, 4T1 cells incubated with AINR displayed much stronger fluorescence intensity than that with F68@TA@Ag, suggesting that the elevation of H_2_O_2_ induced by CDDP could enhance the release of Ag^+^, thus decrease the intracellular Cl^−^.

The level of intracellular free Fe^2+^ determines the stability of Pt–DNA adducts by controlling the activity of DNA repair enzymes. Therefore, we examined the regulation of AINR on intracellular Fe^2+^ (Fig. 4E, and Additional file 1: Figure S15). As expected, a significant decrease of Fe^2+^ was observed in F68@TA, F68@TA@Ag and AINR treated groups, suggesting excellent Fe^2+^ chelation ability of TA involved nanoparticles in tumor cells.

### Self-enhanced antitumor therapy in vitro

**Fig. 5:**
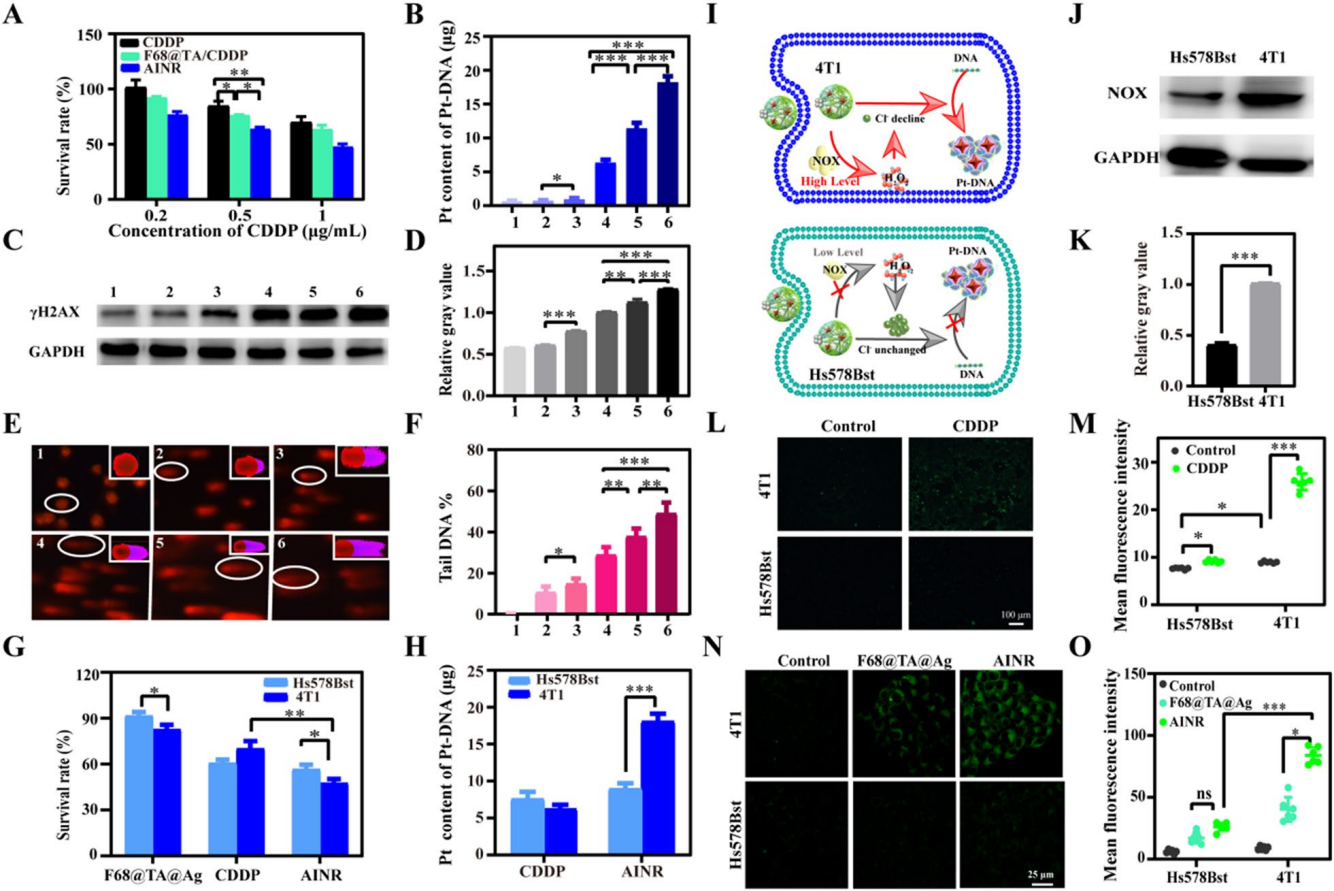
Tumor cell priority of self-enhanced chemotherapy by AINR in vitro. **A** Anti-proliferation ability of different formula containing different concentration of CDDP to 4T1 cells for 24 h (n = 6). **B** Pt content of intracellular DNA extracted from 4T1 treated with different formula by ICP-MS. Before digestion, DNA was fixed as 100 μg (n = 3). **C** Western blotting assays and **D** Gray value analysis of γH2AX and GAPDH in 4T1 cells treated with different CDDP formulation for 24 h. **E** Comet assay images and **F** Corresponding tail DNA percentage analysis of in 4T1 cells treated with different CDDP formulation for 12 h (n = 6). The groups in **B**–**F** were that 1: Control, 2: F68@TA, 3: F68@TA@Ag, 4: CDDP, 5: F68@TA/CDDP, 6: AINR. **G** Survival rate of 4T1 and Hs578Bst cells treated with CDDP, F68@TA@Ag or AINR for 24 h (n = 6) **H** Pt content of intracellular DNA extracted from 4T1 and Hs578Bst cells treated with CDDP or AINR for 24 h by ICP-MS (n = 6). Before digestion, DNA was fixed as 100 μg. **I** Schematic diagram of self-enhanced chemotherapy of AINR with tumor cell priority. **J** Western blotting assays and **K** Gray value analysis of NOX4 and GAPDH in 4T1 and Hs578Bst cells. **L** CLSM images and **M** semi-quantitative analysis of H_2_O_2_ level in 4T1 and Hs578Bst treated with or without CDDP for 18 h (n = 10). **N** CLSM images and **O** semi-quantitative analysis of Cl.^−^ level in 4T1 and Hs578Bst cells treated with F68@TA@Ag or AINR for 12 h (n = 10). In all above experiments, the concentration of CDDP was set as 1 μg/mL. To obtain the approximately same cell uptake, incubation time for 4T1 cells and Hs578Bst cells was postponed to 12 h. The statistical analysis was performed by one-way ANOVA analysis. (ns: no significance, *p < 0.05, ** *p* < 0.01, *** p < 0.001)

The ability of AINR to regulate dual ions lays the foundation for the formation and maintenance of Pt–DNA adducts, then in vitro antitumor effect of AINR was investigated. As displayed in Fig. 5A, both CDDP and CDDP loaded polymer exhibited certain dose-related inhibiting effects on cell growth. Better yet, the significantly highest cytotoxicity was observed after AINR treatment, illustrating that the CDDP chemotherapy could be self-enhanced via AINR (Fig. 5A). Subsequently, to affirm that the self-enhanced inhibition rate induced by AINR was attributed to the Pt–DNA adducts, we explored the amount of Pt–DNA adducts in tumor cells after different treatments. The content of Pt in extracted DNA was evaluated by ICP-MS (Fig. 5B). As expected, AINR induced the most amount of Pt–DNA adducts in all groups. To further investigate whether AINR inhibited cell activity through DNA damage, γH2AX, which could form foci at the location of DNA damage, was chosen as a marker, and the DNA damage was explored by the amount of γH2AX (Fig. 5C, D). Relative gray value of γH2AX in F68@TA/CDDP group was higher than that in CDDP group, which might be ascribed to the down-regulation of Fe^2+^, thus reducing the activity of DNA repair enzymes. AINR induced most DNA damage with the synergic regulation of Cl^−^ and Fe^2+^. The DNA damage was also evaluated by comet assays. As shown in Fig. 5E, F, the tail DNA percentage of 48.9% in AINR group was significantly higher than that in other groups (10.5% in F68@TA group, 14.7% in F68@TA@Ag group, 28.8% in CDDP group and 37.8% in F68@TA/CDDP group), consistent with the γH2AX assays. In a word, AINR could enhance the chemotherapy of CDDP through increasing the amount of Pt–DNA adducts.

Interestingly, we found that the self-enhanced chemotherapy effect induced by AINR might have a tumor cell priority compared with normal cells. The survival rate of 4T1 cells and Hs578Bst cells after treated with AINR was shown in Fig. 5G. The survival rate of 4T1 cells was obviously lower than that of Hs578Bst cells and the amount of Pt–DNA adducts in tumor cells and normal cells were further detected, respectively. As Fig. 5H shown, no significant difference of Pt–DNA adducts was detected between CDDP and AINR in Hs578Bst cells, indicating that AINR did not induce the extra DNA damage. In contrast, the amount of Pt–DNA adducts in the case of AINR group was ~ 3-times higher than that of CDDP group in 4T1 tumor cells.

Combined above phenomenon and related reports, we hypothesis that CDDP can activate NOXs to produce more H_2_O_2_ in tumor cells due to its overexpressed NOX, [28] which helps to dissolve Ag NPs and down-regulate intracellular Cl^−^, thereby promoting the formation of Pt–DNA adducts. While the lower NOXs and low H_2_O_2_ basal content in normal cells lead to insignificant down-regulation of Cl^−^, so there is no obvious self-enhanced chemotherapy effect (Fig. 5I). To prove this, we have investigated NOX4 content, levels of H_2_O_2_, and intracellular Cl^−^ regulation in tumor cells and normal cells. NOX4, one of the most important NOXs, was first detected by western blotting assays. 4T1 cells (breast cancer cell) and Hs578Bst cells (normal breast cells) was selected as the model of tumor cells and normal cells, respectively. The content of NOX4 in 4T1 cells was significantly higher than that in Hs578Bst cells (Fig. 5J, and 5K). Subsequently, specific H_2_O_2_ probe was used to evaluate the level of H_2_O_2_ in both 4T1 and Hs578Bst cells after treated with CDDP. In the case of control group, the green fluorescence in 4T1 cells was obviously higher than that in Hs578Bst cells (Fig. 5L, and M), proving that the initial H_2_O_2_ in 4T1 cells was higher than that in Hs578Bst cells. More importantly, after CDDP treatment, the level of H_2_O_2_ in 4T1 cells showed a significant increase, while little rise in Hs578Bst cells treated with the same concentration of CDDP, indicating the tumor cell selective H_2_O_2_ production.

Increased H_2_O_2_ would contribute to regulation of Cl^−^. Thereby, intracellular Cl^−^ contents in two cells after treated with AINR were detected. To obtain the approximately same cell uptake, incubation time for 4T1 cells and Hs578Bst cells was postponed to 12 h (see detailed methods and Additional file 1: Figure S16 in the Supporting Information). In the case of Hs578Bst cells, no decrease of Cl^−^ was found, while a significant decrease in Cl^−^ was observed in 4T1 cells (Fig. 5N, and O). These results proved that AINR holds preferential self-enhanced chemotherapy for tumor cells, which laid the foundation of attenuated and synergistic treatment of CDDP.

### Deep tumor penetration in 3D tumor spheroid model and solid tumor tissues

**Fig. 6:**
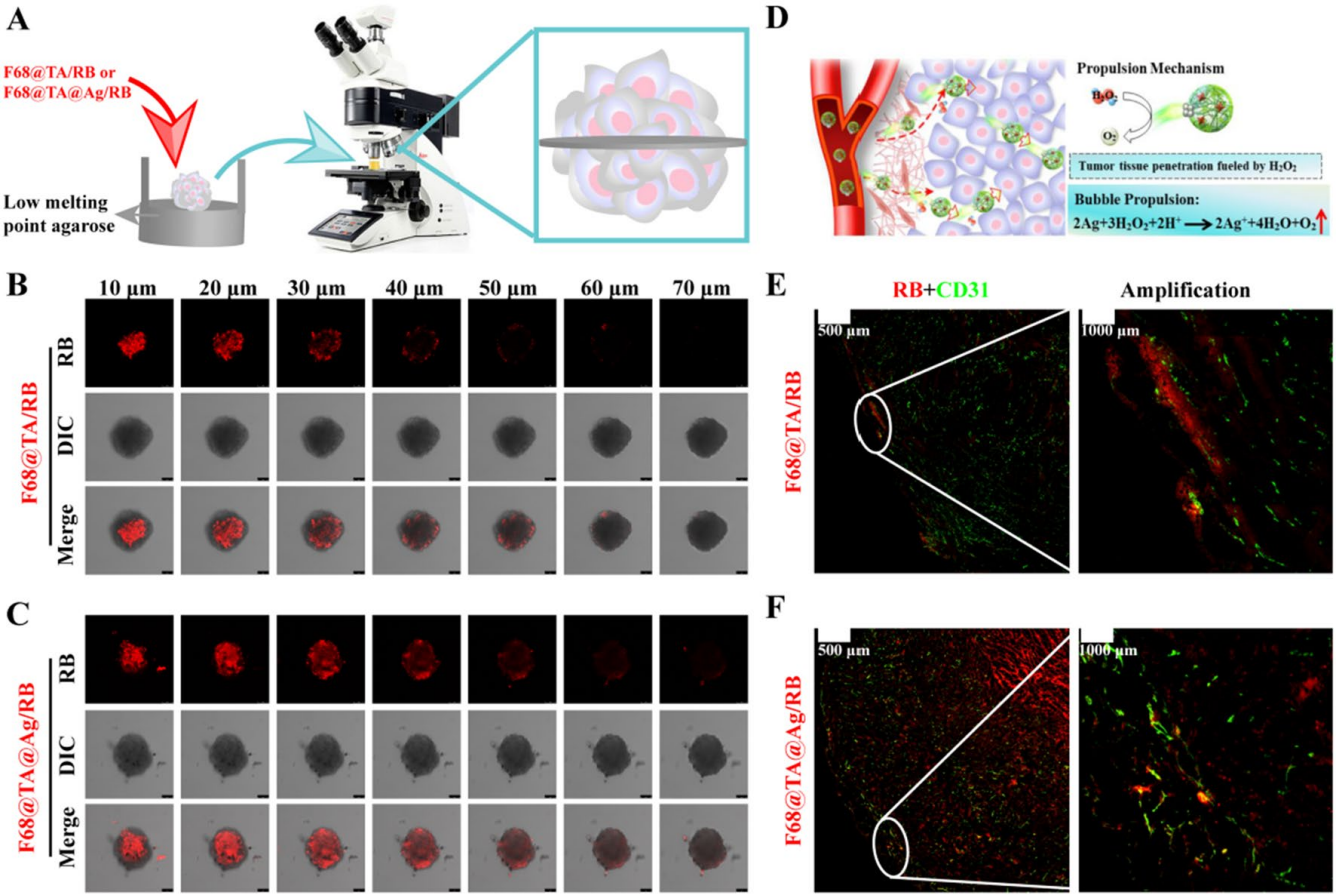
Movability and deep penetration ability of F68@TA@Ag in 3D MCS and tumor tissues. **A** Schematic diagram of investigation on deep penetration capacity in 3D MCS. MCS was constructed on low melting point agarose. Prior to the treatment of RB (10 μg/mL) labeled F68@TA or F68@TA@Ag, 3D MCS was incubated with 100 μM H_2_O_2_ was added for 12 h. CLSM was used to observe RB distribution at different focal planes. **B**, **C** Fluorescence images of MCS in different focal planes after 4T1 cells were incubated with **B** F68@TA/RB and **C** F68@TA@Ag/RB for 12 h. Scale bar: 75 μm. **D** Schematic diagram of investigation on deep penetration capacity in tumor tissues. The movement mechanism was that asymmetrical Ag NPs on F68@TA@Ag could react with H_2_O_2_ at tumor acid environment, leading to O_2_ generation. The reaction equation was presented above. **E**, **F** Immunofluorescent staining of tumor tissues from **E** F68@TA/RB and **F** F68@TA@Ag/RB group. Red color represented nanoparticles and green color represented tumor vessels

Effective penetration of drugs in three-dimensional solid tumors contributes to anti-tumor efficacy [29]. Encouraged by the superior properties of movability fueled by H_2_O_2_, tissue penetration capacity of F68@TA@Ag was elucidated by using three-dimensional multicellular spheroids model (3D MCSs). The penetrations of F68@TA@Ag in MCSs with H_2_O_2_-treatment were observed by CLSM (Fig. 6A). Compared with F68@TA group, red fluorescence (RB) in F68@TA@Ag was located in much more regions of the MCSs even at the depth of 50 μm, indicating that asymmetric nanoparticles (F68@TA@Ag) held the feature of movability at the exist of H_2_O_2_ for deeper tumor tissue penetration (Fig. 6B and 6C). Furthermore, movability of F68@TA@Ag in vivo was explored. 4T1 cells-bearing BALB/C mice were administrated by F68@TA/RB or F68@TA@Ag/RB, and the corresponding tumor tissues were exploited and sliced at 24 h after administration (Fig. 6D). CD31 antibody with green fluorescence was used to label tumor vessels. It can be seen from Fig. 6E, and 6F, compared with F68@TA/RB, most of F68@TA@Ag/RB escaped from tumor vessels and penetrated into the depth of tumor tissues, attributing to the movability of F68@TA@Ag with the help of H_2_O_2_ in tumor environment.

### In vivo biodistribution, antitumor efficiency, and biosafety

**Fig. 7:**
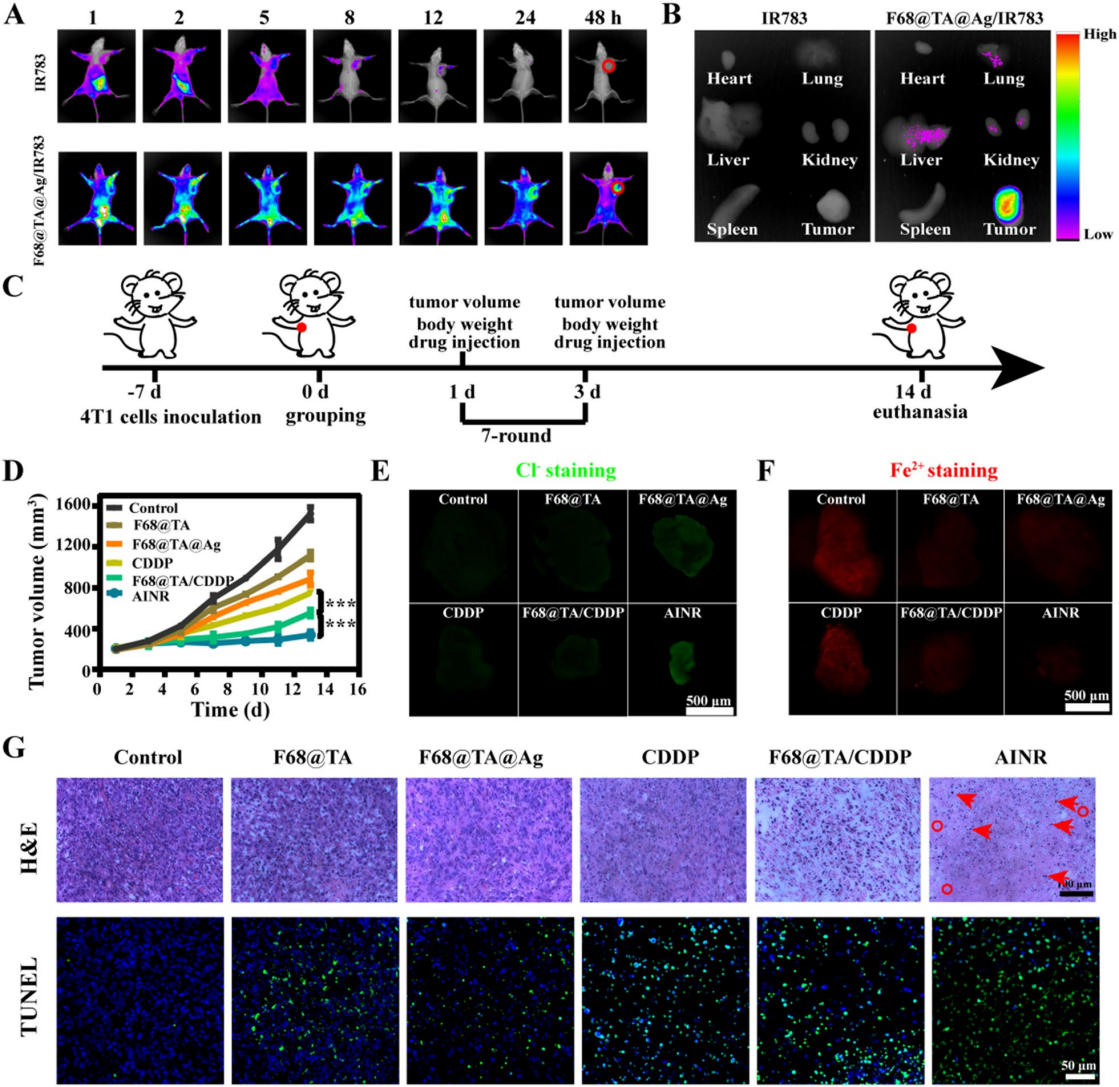
Anti-tumor efficacy of AINR in vivo. **A** Biodistribution of F68@TA@Ag/IR783 in 4T1-bearing BALB/C mice collected by IVIS imaging system at different time point. The red circles referred to tumor tissues. **B** Optical images of visceral organs exfoliated after injection for 48 h. The groups in A-B were a) IR783 and b) F68@TA@Ag/IR783. **C** Schematic diagram of treatment program. **D** The changes of tumor volume with different treatments (n = 5). **E** Representative Cl^−^ staining of tumor tissues from 4T1-bearing BALB/C mice receiving different treatments. **F** Representative Fe^2+^ staining of tumor tissues from 4T1-bearing BALB/C mice receiving different treatments. **G** H&E staining and TUNEL staining of tumor tissues from 4T1-bearing BALB/C mice receiving different treatments, the circle represents nuclear loss, the arrow represents karyopyknosis. In all experiments, concentration of CDDP was set as 2 mg/kg, and statistical analysis was performed by one-way ANOVA analysis. *p < 0.05, **p < 0.01, ***p < 0.001

Encouraged by self-enhanced chemotherapy and H_2_O_2_-driven tissue penetration characteristics of AINR, its antitumor efficiency was explored in vivo. The biodistribution of F68@TA@Ag/IR783 was detected after intravenous injection, and the mice treated with IR783 (a kind of near infrared dyes) were used as the control. As shown in Fig. 7A, compared with free IR783, the circulation time of the F68@TA@Ag/IR783 in vivo was significantly increased. More importantly, F68@TA@Ag/IR783 was accumulated in tumor region efficiently compared to free IR783 even at 48 h post-injection (Fig. 7B), attributing to outstanding stability of F68@TA@Ag/IR783 and the enhanced permeability and retention effect.

Subsequently, the antitumor effect of AINR in vivo was further investigated. Different formulations were injected into tail veins of 4T1 cells-bearing BALB/C mice every two days, respectively. The tumor volume and body weight were recorded during treatment (Fig. 7C). With a 14-d treatment, compared to the control group, the tumors from the mice treated with F68@TA, and F68@TA@Ag showed slight inhibitory effects on growth. It was noteworthy that AINR treatment showed remarkable tumor growth inhibition (Fig. 7D), which was attributed to the H_2_O_2_-driven deep tissue penetration and intracellular ion regulation mediated CDDP self-enhanced chemotherapy. To confirm the mechanism of in vivo self-enhanced effect, tumor tissues from different treated groups were sliced and stained with Cl^−^ probe and Fe^2+^ probe. Cl^−^ level in AINR treated group was significantly lower than that in F68@TA@Ag treated group (Fig. 7E and Additional file 1: Figure S17), indicating the decomposition of Ag NPs into Ag^+^ triggered by CDDP mediated H_2_O_2_ accumulation in the tumor was able to greatly reduce the Cl^−^. In addition, Fe^2+^ level in F68@TA, F68@TA@Ag, F68@TA/CDDP, and AINR were significantly lower than the group that treated by saline and CDDP, indicating excellent free Fe^2+^ chelation ability by TA involved nanoparticles (Fig. 7F, and Additional file 1: Figure S18).

To test the extent of apoptosis of tumor cells treated with different therapies, hematoxylin and eosin (H&E) staining and terminal deoxynucleotidyl transferase dUTP nick end labeling (TUNEL) assay were conducted (Fig. 7G). Large amounts of karyopyknosis (the circle) and the severer nuclear loss (the arrow) was observed in AINR treated group, indicated that tumor cells in the tissue section with AINR treatment were severely damaged. Similarly, more apoptotic cells (green color represented the damaged DNA) were detected in the AINR group by TUNEL assay, which was obviously more than that in other groups. Intuitively, tumor cells in the tissue section with AINR treatment were severely damaged.

Finally, in vivo biosafety of AINR was evaluated. The body weight of mice from CDDP group displayed a significant drop, indicating in vivo toxicity induced by free CDDP. But reasonable weight changes in the normal range were observed in the AINR treated group, indicating negligible systemic toxicity of AINR (Additional file 1: Figure S19). Besides, the major organs (heart, liver, spleen, lung, and kidney) of the mice with different treatments were assessed by H&E staining (Additional file 1: Figure S20). Histological analysis indicated after treatment with free CDDP, renal corpuscle was slightly broken, and renal tubule was swollen, which was attributed to the fact that CDDP is metabolized from kidney as original form, leading to serious nephrotoxicity. All organs in other groups were normal, so it could be inferred there was no obvious systemic toxicity of AINR during the treatment. Moreover, blood was collected for examination of liver function and kidney function (Additional file 1: Figure S21). In CDDP group, the level of ALT was sharply raised, but other indexes of liver function were stable, indicating that detoxification function of liver decreased while protein synthesis capacity remained unchanged. Meanwhile, the contents of CREA, UA, and BUN were all significantly higher than the normal range, suggesting the abnormal kidney function induced by CDDP. The indices of liver and kidney functions in other groups were all within the normal range.

Recently, an increasing attention has been paid to intracellular ion homeostasis, due to its close relationship with various enzyme activities, indispensable role in energy metabolism and the key regulatory mechanism in cell behavior, and the intracellular ion interference becomes a hot topic in tumor treatment [28, 30, 31]. For example, our previous research showed that the efficacy of tumor resistance reversal and immunotherapy could be significant improved through regulating the level of calcium ion in tumor [32, 33]. Specifically, the formation and maintenance of Pt–DNA adducts are affected by the dechlorination of CDDP and the DNA repair enzyme activity, which mainly rely on the intracellular Cl^−^/Fe^2+^ levels. Herein, an **a**symmetric **i**on nanoregulator (AINR) with the ability of regulating intracellular Cl^−^/Fe^2+^ was developed for self-enhanced CDDP chemotherapy. More importantly, AINR held a tumor cell-preferred self-enhanced chemotherapy due to the overexpressed NOXs in tumor cells. With the same therapeutic effect of AINR and CDDP, the dosage of CDDP in AINR was reduced by one-third (Additional file 1: Figure S22), providing a new ion-regulating strategy for enhanced CDDP chemotherapy.

In addition, in vivo delivery efficiency also determines the efficacy of CDDP, and the deep penetration of nanomedicine in tumors has always been a hot topic [34, 35]. To overcome this obstacle, many great efforts have been explored in recent years, such as particle size change strategy, extracellular matrix degradation strategy, etc. [36–38] In this study, during the nanoparticle preparation process, we found the synthesized AINR held a spherical structure with asymmetrically distributed Ag NPs, which performed an autonomous movable property as a nanomotor. With the ability of self-propelled movement, AINR could overcome the extracellular matrix barrier and penetrate into deeper tumor. Interestingly, different from other nanomotors that were usually prepared by template-directed electrodeposition process [39–41], AINR with asymmetric structure could be prepared through one step in situ reduction in solution environment. The simple preparation process of AINR is expected to provide a deep penetrating treatment platform for solid tumors and inflammatory environment.

## Conclusions

In summary, for the first time, we successfully synthesized a cisplatin loading nanomotor based janus structured Ag-polymer (AINR) with autonomous penetration and dual-ions (Fe^2+^/Cl^−^) regulated ability for enhanced CDDP chemotherapy. AINR held a significant movable ability fueled by H_2_O_2_ in tumor issue, which can drive drug to penetrate into deeper tumor. After being efficiently internalized by tumor cells, AINR presented unique self-enhanced CDDP chemotherapy: CDDP induced the production of H_2_O_2_ through specific activation of NOXs; the generated H_2_O_2_ in turn promoted the accumulation of intracellular Pt–DNA adducts (formation and maintenance) with effective modulation of intracellular Cl^−^ and Fe^2+^. It is noteworthy that overexpressed NOXs in tumor cells provided selectivity for self-enhanced CDDP chemotherapy. Finally, AINR resulted in 80% of tumor growth inhibition in a 4T1 mammary adenocarcinoma model through harnessing the cascade chemotherapeutic barriers. This study offered a systematic strategy of ion regulation, autonomous movement for enhanced cisplatin-based tumor treatment.

## Supplementary Information

**Additional file 1: Figure S1**. DLS analysis of F68@TA prepared by different mass ratio of F68 and TA. (n=3). **Figure S2**. TEM images of F68@TA@Ag NPs prepared with different content of AgNO_3_. **Figure S3**. (A) Size distributions and (B) zeta potentials of F68@TA, F68@TA/CDDP, and AINR solutions. **Figure S4**. Tyndall Effects of F68@TA/CDDP solution at different pHs for 1 h. **Figure S5**. DLS analysis of F68@TA/CDDP treated with PBS at different pHs for 48 h. **Figure S6**. HPLC chromatograms of different solvents and corresponsive CDDP (40 μg/mL) solution for 2 d. **Figure S7**. (A) Graphs and (B) UV-vis spectrum of F68@TA@Ag solutions with different concentration of H_2_O_2_. **Figure S8**. TBE-PAGE gel electrophoregram of DNA. 1: unmethylated single strand DNA; 2: unmethylated single strand DNA cut by restriction enzyme DnpII; 3: m1A methylated DNA; 4: m1A DNA sheared by DnpII. **Figure S9**. A) The tracking paths of F68@TA@Ag over 20 s in 0, 2.5, 5, 10, 25 and 50 mM H_2_O_2_. B) Average MSD versus time interval (Δt) analyzed from tracking trajectories. C) Corresponding diffusion coefficient values of F68@TA@Ag at various concentration of H_2_O_2_. (n=30). **Figure S10**. Fluorescence emission spectrums of FITC and RB dissolved in PBS at different pHs. **Figure S11**. A) Flow cytometry analysis and B) Fluorescence semi-quantitative analysis of 4T1 cells treated with F68@TA/RB for different time. **Figure S12**. A) CLSM images and B) Fluorescence semi-quantitative analysis of 4T1 cancer cells after treatments with F68@TA/RB (RB: 10 μg/mL) for different time. Scale bar: 25 μm. **Figure S13**. Fluorescence semi-quantitative analysis of H_2_O_2_ in 4T1 cells after treatment with different concentration of CDDP. **Figure S14**. Fluorescence semi-quantitative analysis of Cl^-^ in 4T1 cells after treatment with CDDP, F68@TA, F68@TA@Ag and AINR. **Figure S15**. Fluorescence semi-quantitative analysis of Fe^2+^ in 4T1 cells after treatment with CDDP, F68@TA, F68@TA@Ag and AINR. **Figure S16**. Flow cytometry analysis of the fluorescence intensity in A) Hs578Bst cells and B) 4T1 cells treated with F68@TA/RB (RB: 5 μg/mL) for different time. C) Fluorescence semi-quantitative analysis of 4T1 cells and Hs578Bst cells. **Figure S17**. Fluorescence semi-quantitative analysis of Cl^-^ in tumor tissues. **Figure S18**. Fluorescence semi-quantitative analysis of Fe^2+^ in tumor tissues. **Figure S19**. Body weight changes of different groups. (n = 5). **Figure S20**. H&E staining of tissues exfoliated from different groups. **Figure S21**. Serum biochemical index. A) Liver function and B) Kidney function index of mice treated with different formulation. 1: Control, 2: F68@TA, 3: F68@TA@Ag, 4: CDDP, 5: F68@TA/CDDP, 6: AINR, and concentration of CDDP was set as 1 μg/mL. **Figure S22**. The changes of tumor volume of 4T1 cell bearing mice with the treatment of AINR (CDDP: 2 mg/kg), 2 mg/kg CDDP, and 3 mg/kg CDDP. (n=5). **Table S1**. The DNA sequences of unmethylated DNA and methylated DNA.

**Additional file 2: Video S1**. O_2_ generation during the reaction of F68@TA@Ag nanoparticles with H_2_O_2_

**Additional file 3: Video S2**. Tracking trajectories of F68@TA@Ag in different concentration of H_2_O_2 _

## Data Availability

The datasets used and/or analyzed during the current study are available from the corresponding author on reasonable request.
