# Supplementary material for: Boosting cisplatin chemotherapy by nanomotor-enhanced tumor penetration and DNA adducts formation
Source: J Nanobiotechnology. 2022 Sep 29;20:429. doi: 10.1186/s12951-022-01622-3 (PMC9523964; doi:10.1186/s12951-022-01622-3)
Supplement: Supplementary file 1 — Additional file 1: Figure S1. DLS analysis of F68@TA prepared by different mass ratio of F68 and TA. (n=3). Figure S2. TEM images of F68@TA@Ag NPs prepared with different content of AgNO3. Figure S3. (A) Size distributions and (B) zeta potentials of F68@TA, F68@TA/CDDP, and AINR solutions. Figure S4. Tyndall Effects of F68@TA/CDDP solution at different pHs for 1 h. Figure S5. DLS analysis of F68@TA/CDDP treated with PBS at different pHs for 48 h. Figure S6. HPLC chromatograms of different solvents and corresponsive CDDP (40 μg/mL) solution for 2 d. Figure S7. (A) Graphs and (B) UV-vis spectrum of F68@TA@Ag solutions with different concentration of H2O2. Figure S8. TBE-PAGE gel electrophoregram of DNA. 1: unmethylated single strand DNA; 2: unmethylated single strand DNA cut by restriction enzyme DnpII; 3: m1A methylated DNA; 4: m1A DNA sheared by DnpII. Figure S9. A) The tracking paths of F68@TA@Ag over 20 s in 0, 2.5, 5, 10, 25 and 50 mM H2O2. B) Average MSD versus time interval (Δt) analyzed from tracking trajectories. C) Corresponding diffusion coefficient values of F68@TA@Ag at various concentration of H2O2. (n=30). Figure S10. Fluorescence emission spectrums of FITC and RB dissolved in PBS at different pHs. Figure S11. A) Flow cytometry analysis and B) Fluorescence semi-quantitative analysis of 4T1 cells treated with F68@TA/RB for different time. Figure S12. A) CLSM images and B) Fluorescence semi-quantitative analysis of 4T1 cancer cells after treatments with F68@TA/RB (RB: 10 μg/mL) for different time. Scale bar: 25 μm. Figure S13. Fluorescence semi-quantitative analysis of H2O2 in 4T1 cells after treatment with different concentration of CDDP. Figure S14. Fluorescence semi-quantitative analysis of Cl- in 4T1 cells after treatment with CDDP, F68@TA, F68@TA@Ag and AINR. Figure S15. Fluorescence semi-quantitative analysis of Fe2+ in 4T1 cells after treatment with CDDP, F68@TA, F68@TA@Ag and AINR. Figure S16. Flow cytometry analysis of the fluorescence intensity [file 12951_2022_1622_MOESM1_ESM.docx]

**Supporting information**

**Boosting cisplatin chemotherapy by nanomotor-enhanced tumor penetration and DNA adducts formation**

Lihua Xu^a^, Kaixiang Zhang^e^, Xing Ma^d^, Yingying Li^e^, Yajie Jin^e^, Chenglin Liang^e^, Yong Wang^d^, Wendi Duan^d^, Hongling Zhang^e^, Zhenzhong Zhang^e^, Jinjin Shi^e^, Junjie Liu^e^, Yunlong Wang ^c,^*, Wentao Li ^b,^*

^a^ National Center for International Research in Cell and Gene Therapy, Sino-British Research Center for Molecular Oncology, Academy of Medical Sciences, Zhengzhou University, Zhengzhou 450001, China.

^b^ People’s Hospital of Henan University, Zhengzhou 450003, Henan, China

^c^ Henan Bioengineering Research Center, Zhengzhou, Henan, China

^d^ School of Materials Science and Engineering & Flexible Printed Electronic Technology Center, Harbin Institute of Technology (Shenzhen), Shenzhen 518055, China.

^e^ School of Pharmaceutical Sciences, Zhengzhou University, Zhengzhou 450001, China

**1 Experimental section**

**1.1 Cell culture**

4T1 (mouse breast cancer cells) and Hs578Bst (mouse normal mammary cells) cell lines were purchased from Cell Bank of Chinese Academy of Science (Shanghai, P. R. China) and cultured using RPMI 1640 medium (Solarbio, China) supplemented with 10% fetal bovine serum (FBS). Cells were seeded in the cell culture flask at 37 ℃ and 5% CO_2_ in a humidified incubator.

**1.2. Cellular uptake and lysosomal distribution of F68@TA/RB in 4T1 cells**

To study the distribution behavior of F68@TA in 4T1 cells especially in lysosomes, F68@TA/RB was prepared according to the synthesis process of F68@TA/CDDP. 4T1 cells was seeded in a 6-well plate with the density of 3×10^5^ cells per well. After cells adhered to the plate, culture medium containing F68@TA/RB (RB: 10 μg/mL) was added and co-incubated with cells for different time (0.5, 1, 2, 3, 4, and 6 h). Then flow cytometry (Accuri C6) and CLSM (TCS SP8 STED) were used to detect the cellular uptake of F68@TA/RB for different groups.

In addition, lysosomes were marked by lysosomal tracker with green fluorescence. Colocation efficiency of F68@TA/RB and lysosomes at different incubation time points (2, 4, and 7 h) was evaluated by CLSM and analyzed by Image J.

**1.3. Tumor cell selective self-amplification of MINR**

For tumor cell selective self-amplification studies, 4T1 cells were used as the tumor cell model, and Hs578Bst cells were used as the healthy cell model. The equivalent concentration of CDDP was 1 μg/mL.

*NOX4 level detection in 4T1 and Hs578Bst cells:* 4T1 and Hs578Bst cells in logarithmic phase were collected and lysed for total proteins. Then western blotting assays were conducted, in which NOX4 and GAPDH antibody was used. After exposure, the relative gray value of NOX4 and GAPDH was conducted for semi-quantitative analysis.

*H_2_O_2_ level detection in 4T1 and Hs578Bst cells:* 4T1 and Hs578Bst cells were seeded in 6-well plates, and treated with or without CDDP for 18 h. Then BES-H_2_O_2_-Ac probe was used to visualize the intracellular H_2_O_2_ level. Fluorescence semi-quantitative analysis was conducted by Image J.

*Cellular uptake of F68@TA@Ag in 4T1 and Hs578Bst cells:* 4T1 and Hs578Bst cells were seeded in 6-well plates overnight and incubated with F68@TA@Ag/RB (RB: 5 μg/mL) for different time (0, 4, and 12 h). After incubation, cells were washed by PBS twice and collected for flow cytometry test.

*Cl^-^ level detection in 4T1 and Hs578Bst cells:* 4T1 and Hs578Bst cells were seeded in 6-well plates, and treated with F68@TA@Ag, or AINR for 12 h. Then MQAE probe was used to visualize the intracellular Cl^-^ level. Fluorescence semi-quantitative analysis was conducted by Image J.

*Pt content detection in 4T1 and Hs578Bst cells:* 4T1 and Hs578Bst cells were seeded in 6-well plates, and treated with CDDP, or AINR for 24 h. After incubation, cells were used for Pt content detection.

*Cytotoxicity in 4T1 and Hs578Bst cells:* 4T1 and Hs578Bst cells were seeded in 96-well plates, and treated with CDDP (1 μg/mL), F68@TA@Ag, or AINR for 24 h. CCK8 was used to evaluate the survival rate of 4T1 and Hs578Bst cells after different treatment.

**1.4. Establishment of animal models**

Female BALB/c mice (5−6 weeks, 16 ± 2 g, No. DW2020060014) was purchased from Experimental Animal Center of Henan Province (Zhengzhou, China). All animal experiments were performed in compliance with the Institutional Animal Care and Use Committee of Zhengzhou University (ZZU-19211-1-6). Accreditation number of the animal laboratory is SCXK (YU) 2018-0004. The 4T1 cells (4×10^6^) were subcutaneously inoculated on the right forelimb of BALB/C mice. The tumor volume of mice was measured using calipers every two days. The tumor volumes were calculated by the following formulation: tumor volume (mm^3^) = (tumor length) × (tumor width)^2^)/2. 4T1 cells bearing mice were used for animal experiments when the tumor volume reached 100-200 mm^3^. The mice treated with CDDP (3 mg/kg) were set as positive control.

**2 Supporting Results**


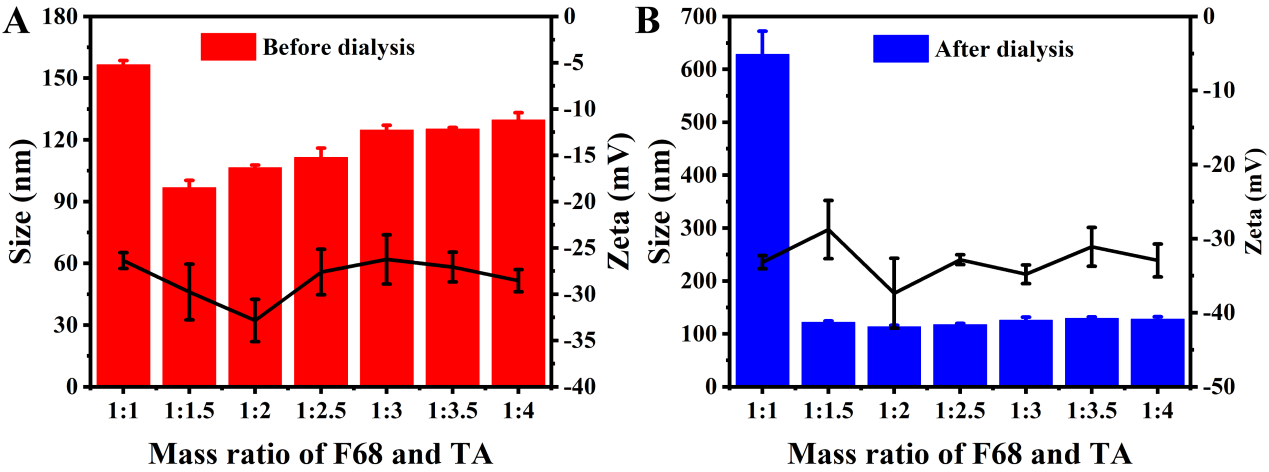


**Figure S1.** DLS analysis of F68@TA prepared by different mass ratio of F68 and TA. (n=3)


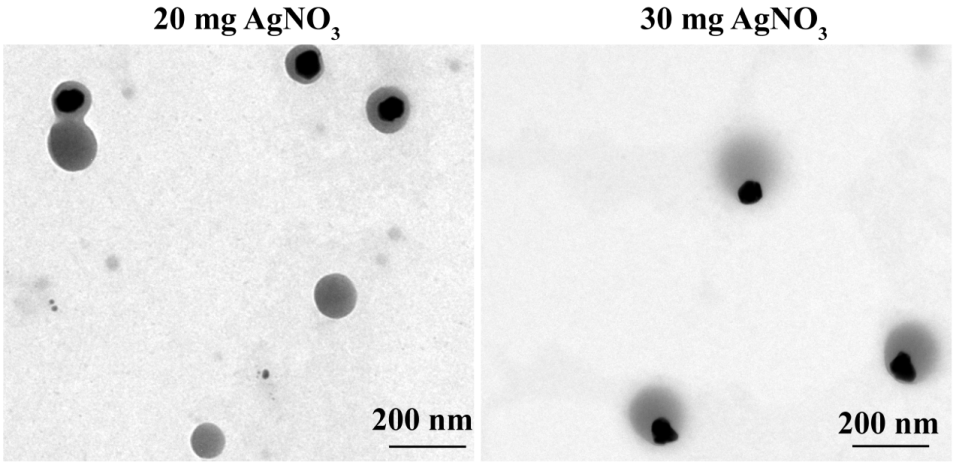


**Figure S2.** TEM images of F68@TA@Ag NPs prepared with different content of AgNO_3_.


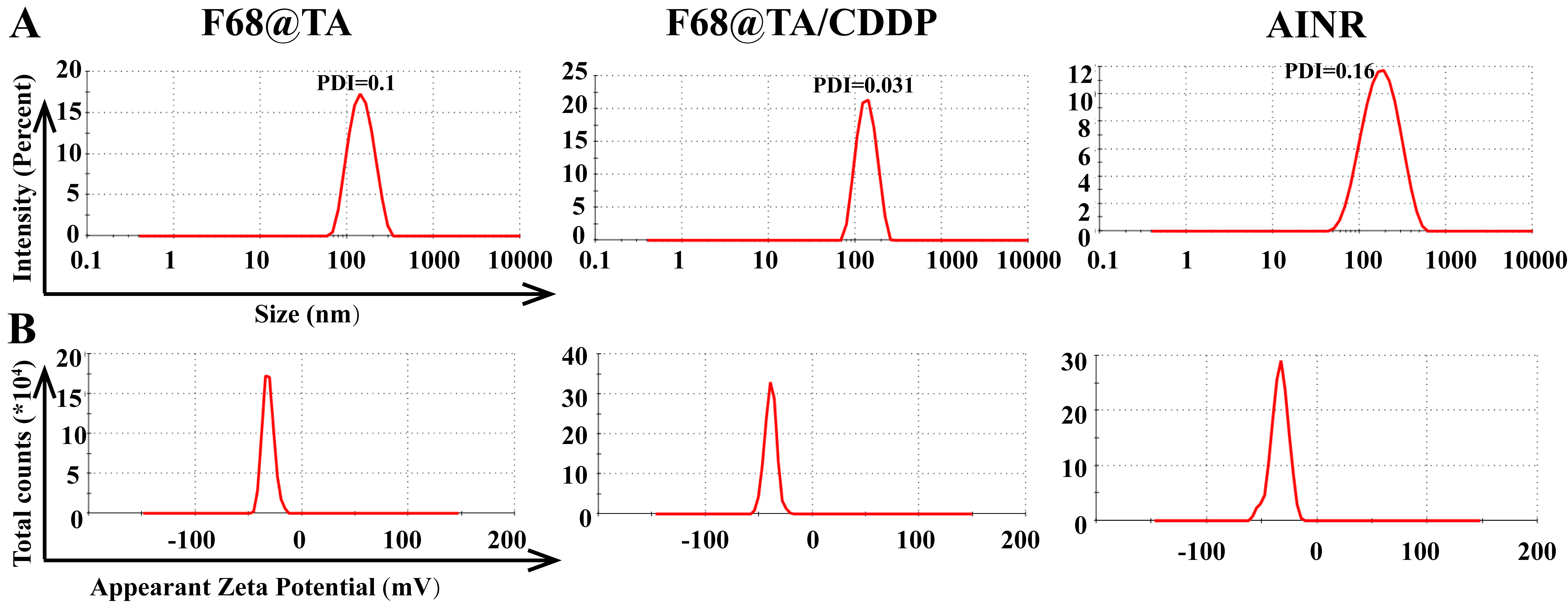


**Figure S3.** (A) Size distributions and (B) zeta potentials of F68@TA, F68@TA/CDDP, and AINR solutions.


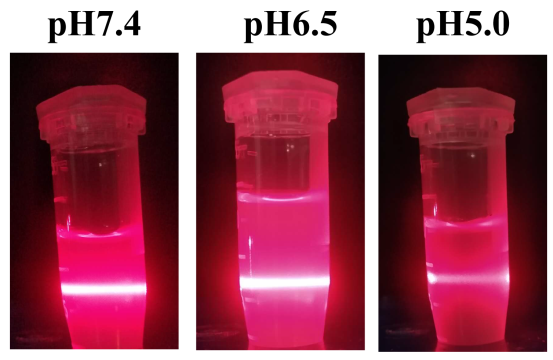


**Figure S4.** Tyndall Effects of F68@TA/CDDP solution at different pHs for 1 h.


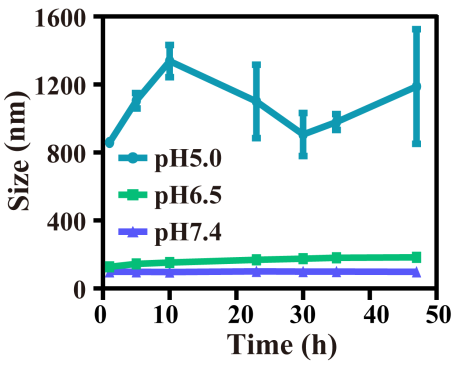


**Figure S5.** DLS analysis of F68@TA/CDDP treated with PBS at different pHs for 48 h


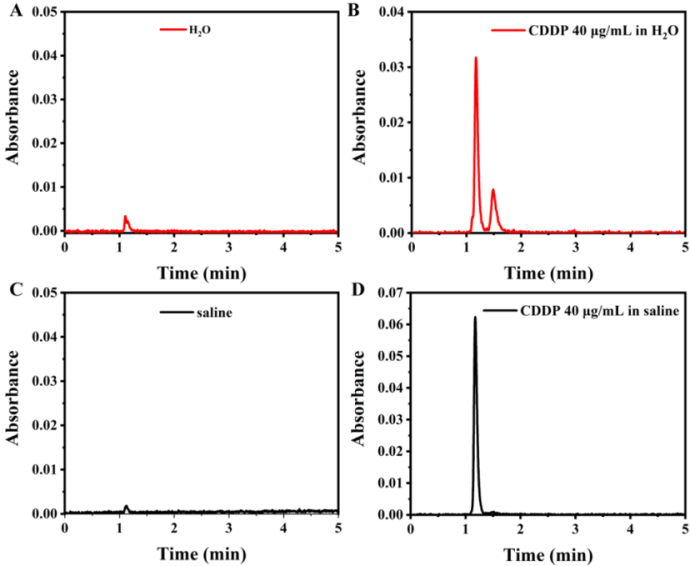


**Figure S6.** HPLC chromatograms of different solvents and corresponsive CDDP (40 μg/mL) solution for 2 d.


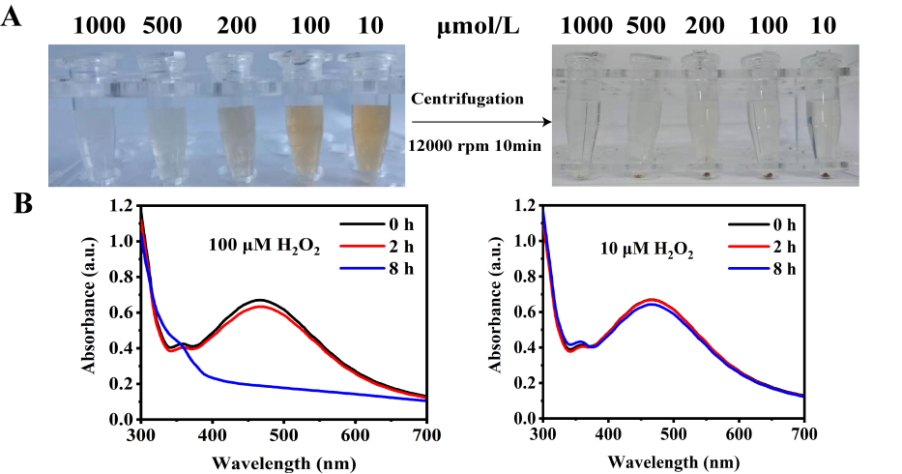


**Figure S7.** (A) Graphs and (B) UV-vis spectrum of F68@TA@Ag solutions with different concentration of H_2_O_2._

**Table S1** The DNA sequences of unmethylated DNA and methylated DNA

| DNA | sequence |
| --- | --- |
| Unmethylated DNA | ATTGCCATTCTCGATAGGTCCGGTCAAAC-TAGACGA |
| Methylated DNA | ATTGCCATTCTCGATAGG(m^1^A)TCCGGTCAAACCTAGACGA |


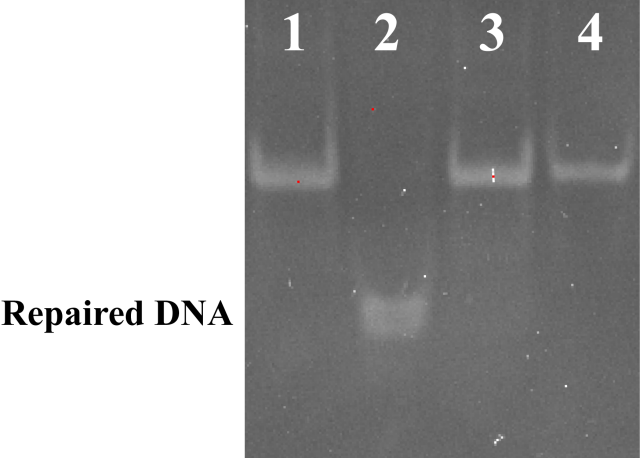


**Figure S8.** TBE-PAGE gel electrophoregram of DNA. 1: unmethylated single strand DNA; 2: unmethylated single strand DNA cut by restriction enzyme DnpⅡ; 3: m1A methylated DNA; 4: m1A DNA sheared by DnpⅡ.


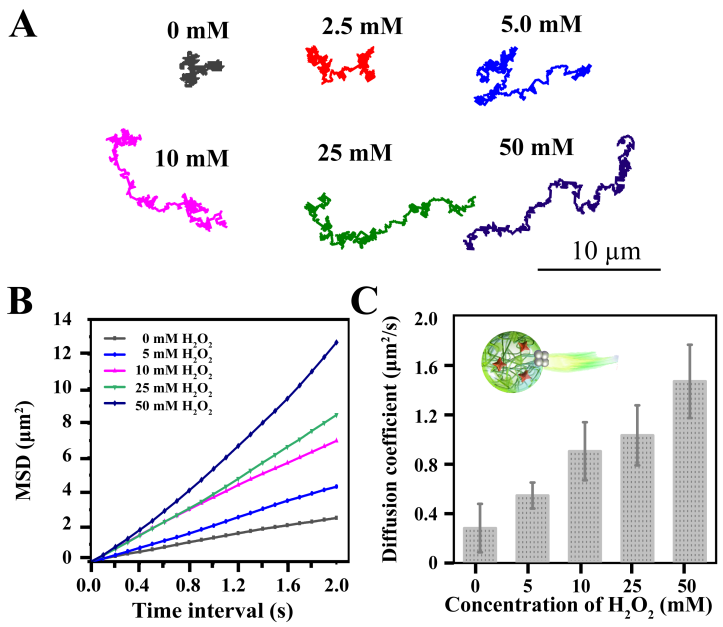


**Figure S9.** A) The tracking paths of F68@TA@Ag over 20 s in 0, 2.5, 5, 10, 25 and 50 mM H_2_O_2_. B) Average MSD versus time interval (Δt) analyzed from tracking trajectories. C) Corresponding diffusion coefficient values of F68@TA@Ag at various concentration of H_2_O_2_. (n=30).

**
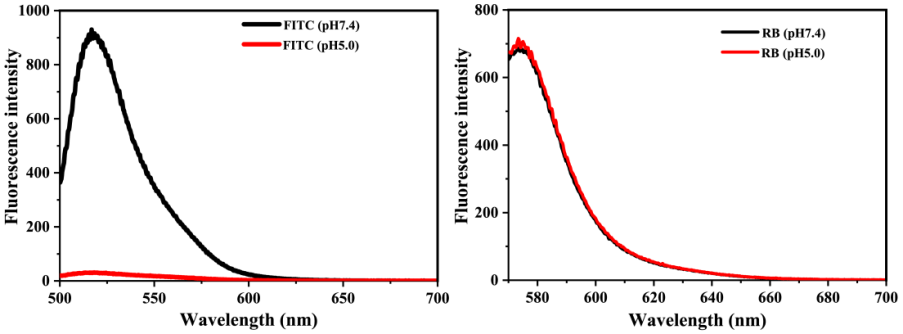
**

**Figure S10.** Fluorescence emission spectrums of FITC and RB dissolved in PBS at different pHs.

**
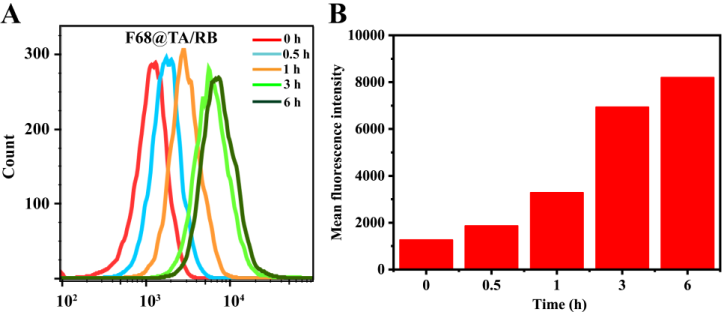
**

**Figure S11.** A) Flow cytometry analysis and B) Fluorescence semi-quantitative analysis of 4T1 cells treated with F68@TA/RB for different time.


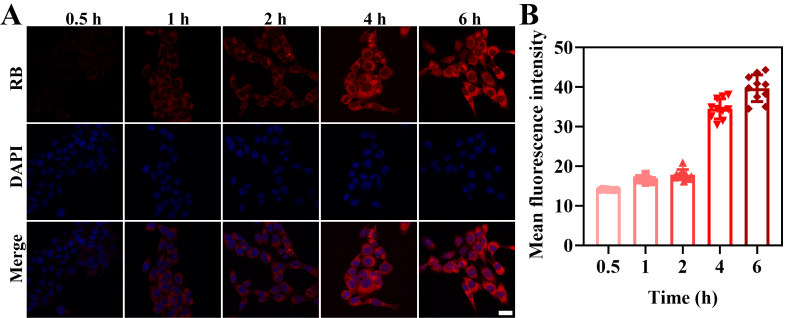


**Figure S12.** A) CLSM images and B) Fluorescence semi-quantitative analysis of 4T1 cancer cells after treatments with F68@TA/RB (RB: 10 μg/mL) for different time. Scale bar: 25 μm.


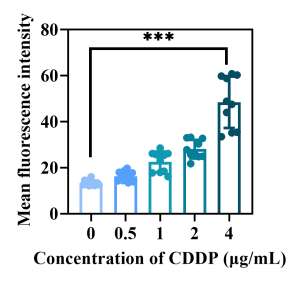


**Figure S13.** Fluorescence semi-quantitative analysis of H_2_O_2_ in 4T1 cells after treatment with different concentration of CDDP.


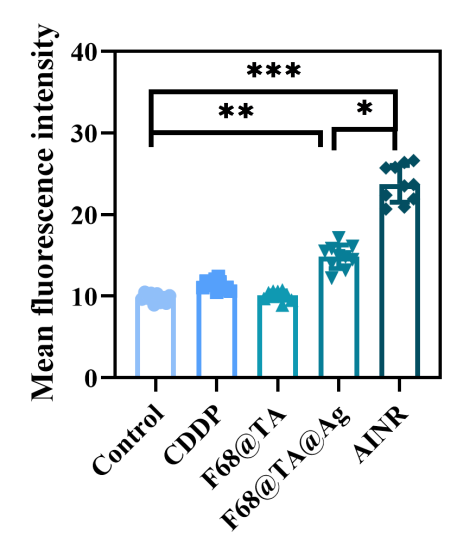


**Figure S14.** Fluorescence semi-quantitative analysis of Cl^-^ in 4T1 cells after treatment with CDDP, F68@TA, F68@TA@Ag and AINR.


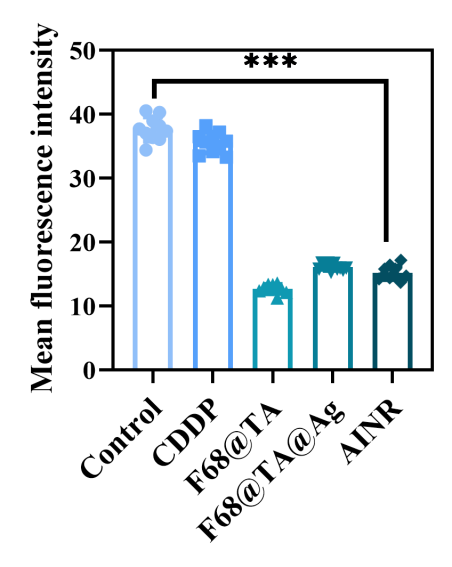


**Figure S15.** Fluorescence semi-quantitative analysis of Fe^2+^ in 4T1 cells after treatment with CDDP, F68@TA, F68@TA@Ag and AINR.


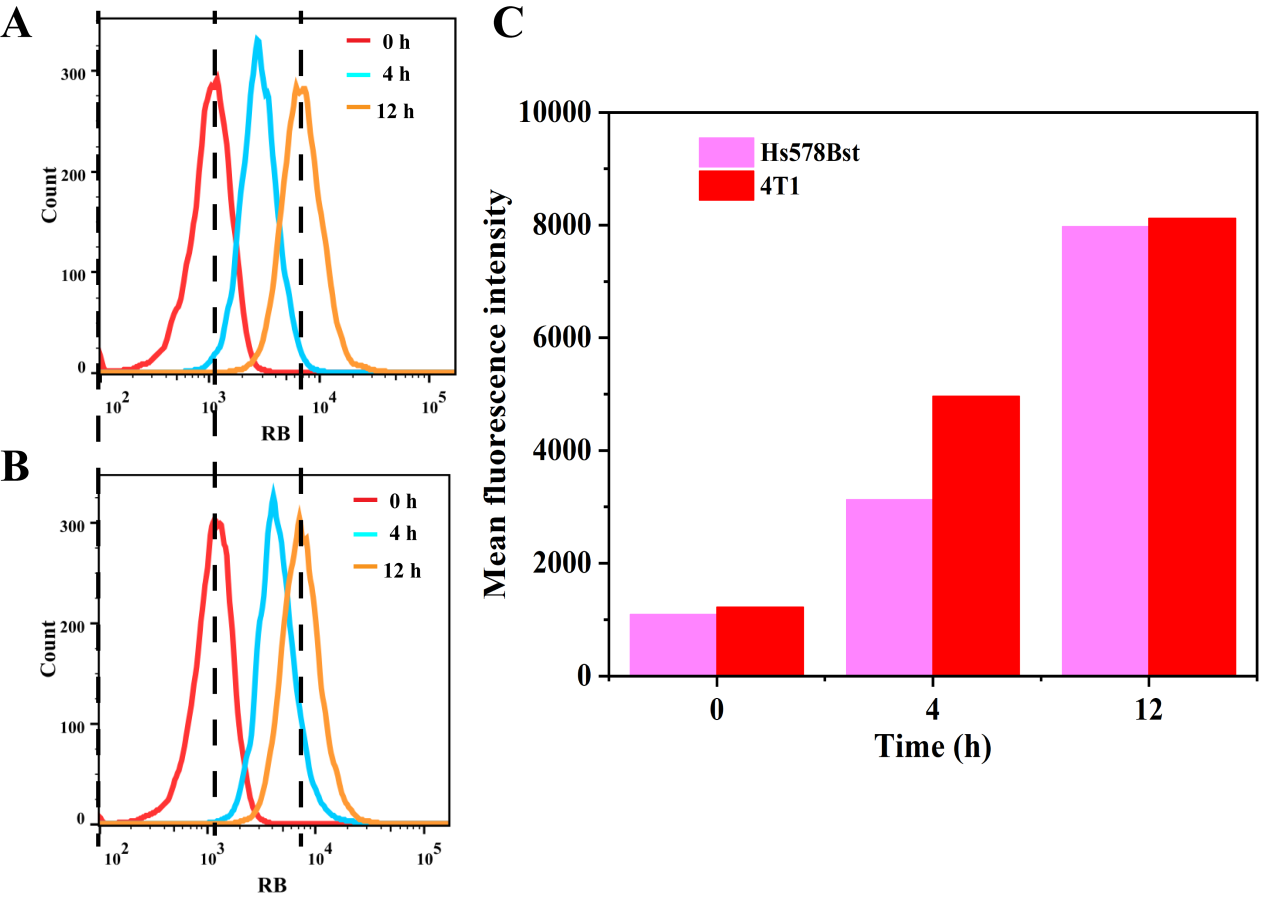


**Figure S16.** Flow cytometry analysis of the fluorescence intensity in A) Hs578Bst cells and B) 4T1 cells treated with F68@TA/RB (RB: 5 μg/mL) for different time. C) Fluorescence semi-quantitative analysis of 4T1 cells and Hs578Bst cells.


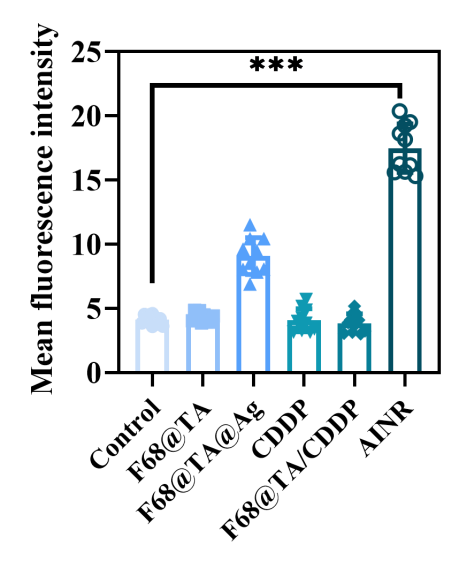


**Figure S17.** Fluorescence semi-quantitative analysis of Cl^-^ in tumor tissues.


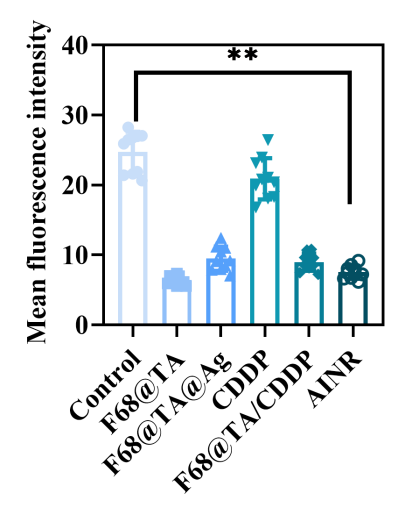


**Figure S18.** Fluorescence semi-quantitative analysis of Fe^2+^ in tumor tissues.





**Figure S19.** Body weight changes of different groups. (n = 5)


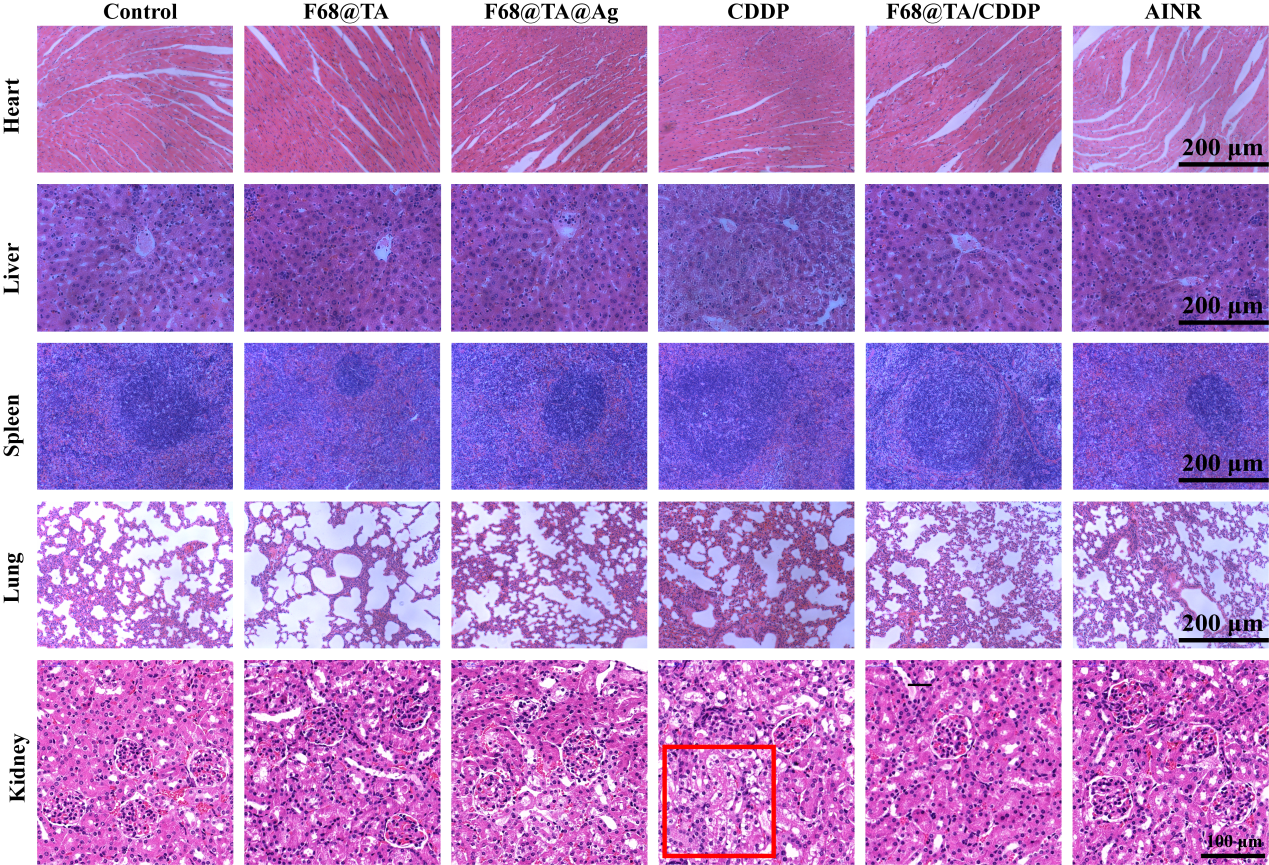


**Figure S20.** H&E staining of tissues exfoliated from different groups.


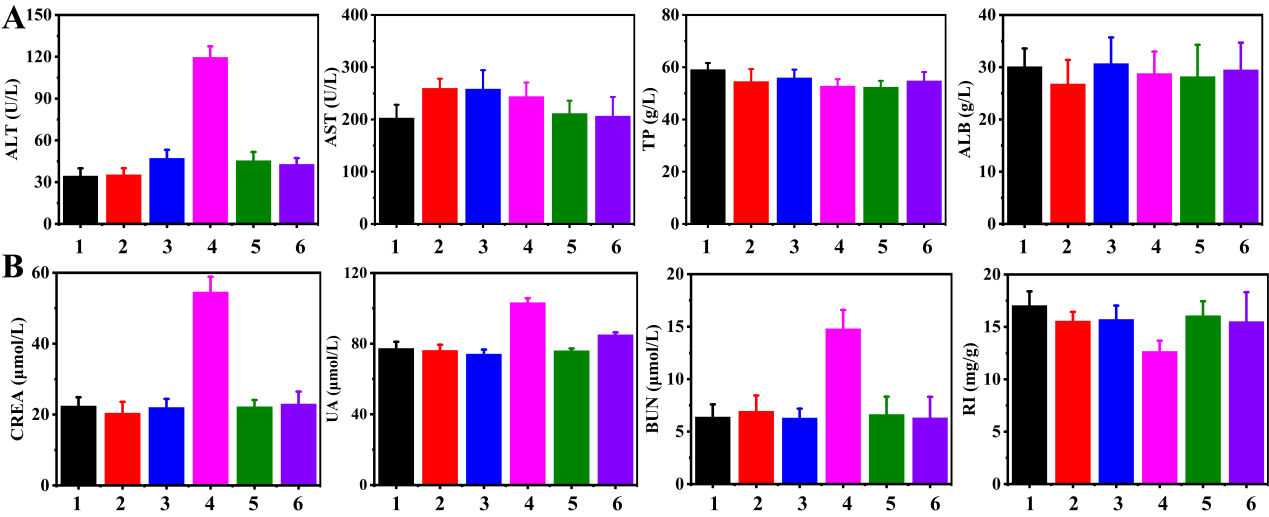


**Figure S21.** Serum biochemical index. A) Liver function and B) Kidney function index of mice treated with different formulation. 1: Control, 2: F68@TA, 3: F68@TA@Ag, 4: CDDP, 5: F68@TA/CDDP, 6: AINR, and concentration of CDDP was set as 1 μg/mL.


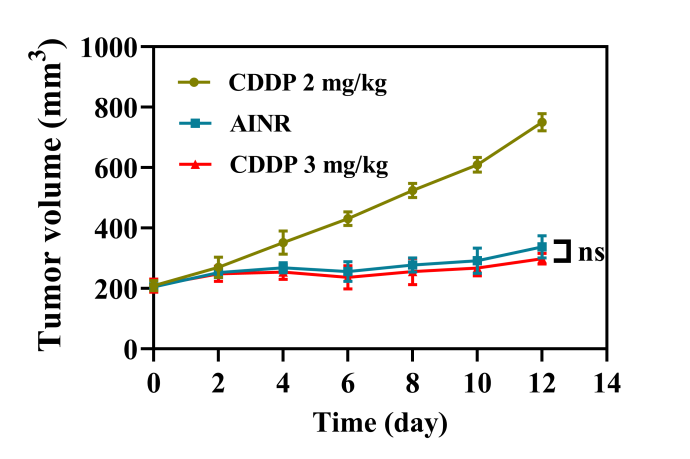


**Figure S22.** The changes of tumor volume of 4T1 cell bearing mice with the treatment of AINR (CDDP: 2 mg/kg), 2 mg/kg CDDP, and 3 mg/kg CDDP. (n=5)
